# Cpx-signalling facilitates Hms-dependent biofilm formation by *Yersinia pseudotuberculosis*

**DOI:** 10.1038/s41522-022-00281-4

**Published:** 2022-03-29

**Authors:** Dharmender K. Gahlot, Sun N. Wai, David L. Erickson, Matthew S. Francis

**Affiliations:** 1 Department of Molecular Biology, Umeå University, Umeå, Sweden; 2 Umeå Centre for Microbial Research, Umeå University, Umeå, Sweden; 3 The Laboratory for Molecular Infection Medicine, Umeå University, Umeå, Sweden; 4 Department of Microbiology and Molecular Biology, Brigham Young University, Provo, UT USA

**Keywords:** Biofilms, Microbial genetics

## Abstract

Bacteria often reside in sessile communities called biofilms, where they adhere to a variety of surfaces and exist as aggregates in a viscous polymeric matrix. Biofilms are resistant to antimicrobial treatments, and are a major contributor to the persistence and chronicity of many bacterial infections. Herein, we determined that the CpxA-CpxR two-component system influenced the ability of enteropathogenic *Yersinia pseudotuberculosis* to develop biofilms. Mutant bacteria that accumulated the active CpxR~P isoform failed to form biofilms on plastic or on the surface of the *Caenorhabditis elegans* nematode. A failure to form biofilms on the worm surface prompted their survival when grown on the lawns of *Y. pseudotuberculosis*. Exopolysaccharide production by the *hms* loci is the major driver of biofilms formed by *Yersinia*. We used a number of molecular genetic approaches to demonstrate that active CpxR~P binds directly to the promoter regulatory elements of the *hms* loci to activate the repressors of *hms* expression and to repress the activators of *hms* expression. Consequently, active Cpx-signalling culminated in a loss of exopolysaccharide production. Hence, the development of *Y. pseudotuberculosis* biofilms on multiple surfaces is controlled by the Cpx-signalling, and at least in part this occurs through repressive effects on the Hms-dependent exopolysaccharide production.

## Introduction

*Yersinia pseudotuberculosis* (*Yptb*) is a common foodborne pathogen that most often causes mild and self-limiting gastrointestinal illnesses^1^. This bacteria is the progenitor of the deadly zoonotic pathogen, *Yersinia pestis* (*Ype*), the causative agent of the devasting diseases, bubonic and septicemic plague^2,3^. The two species share a genomic identity of ~97%^4^, yet are distinguishable by the diverse repertoires of pathophysiological factors that they can encode^5^.

One of the most crucial pathophysiological features associated with many bacteria is the ability to form biofilms. Biofilms are a viscous polymeric matrix of bacterial communities capable of growth on abiotic and biotic surfaces, and which are inherently resistant to antimicrobial agents that causes significant problems for the clinicians trying to treat the bacterial infections^6–8^. Regardless of the species, *Yersinia* survival in the environment or during an infection is often dependent upon the ability to develop a biofilm^9–11^.

A core component of *Yersinia* biofilm is the extracellular matrix material termed exopolysaccharide (EPS). The production and transport of EPS is controlled by the chromosomal *hms* loci in *Yptb*^9,12^ and *Ype*^12–24^. Yet recent ‘omics’ based applications using *Yptb*^25,26^ and *Ype*^25,27,28^ models has uncovered many additional structural factors, metabolic pathways and integrated regulatory circuits involved in *Yersinia* biofilm development. It is now known that the quorum sensing, RovA-RovM and RcsA-RcsB regulatory cascades are common processes controlling *Yersinia* biofilms, albeit they have contrasting affects in *Yptb*^26,29–32^ compared to *Ype*^20,28,31–35^. These observations are consistent with *Yptb* and *Ype* having evolved in different environments. By extension, it is not surprising that the different species and even serotypes within species have also co-opted specific regulatory circuitry to contribute to biofilm formation. For example, RpoS^36^, BarA/UvrY^37^, motility^38^ and a type VI secretion system^25^ are associated with *Yptb* biofilm development. On the other hand, various factors that fine-tune bacterial surface characteristics^39–41^, as well as metabolic adaptation and the function of small regulatory RNAs^42–48^ are all associated with *Ype* biofilm development.

From earlier work on *Yptb*, the classical CpxAR two-component signalling system is involved in modulating the regulatory cascade outputs of RovA-RovM^49,50^ and the Rcs phosphorelay system^51^. These couplings suggests that Cpx-signalling might also be involved in controlling aspects of biofilm development. The CpxAR system has historically been implicated in maintaining the integrity of the bacterial envelope during exposure to extracytoplasmic stresses^52^. CpxA functions as both a sensor kinase and a phosphatase for the cognate response regulator, CpxR. In response to particular extracyoplasmic stresses, CpxA is first an autokinase and then a phosphoryl donor to CpxR. The active phosphorylated CpxR (CpxR~P) isoform subsequently serves as a transcription activator or repressor for the controlled expression of many genes^53–56^. In the absence of stress, CpxA phosphatase activity predominates, and this serves to dephosporylate CpxR**~**P keeping it inactive. While the mechanisms of Cpx-signalling have been mostly established in a laboratory strain of *Escherichia coli*, it has emerged recently that this pathway is an important molecular switch controlling virulence gene expression in pathogenic *Yersinia* and many other clinically and agriculturally relevant Gram-negative bacterial pathogens^57^.

Establishment of cohesive biofilms require bacteria to sense the surfaces they encounter^58^. Exactly how this might occur is largely mysterious. There is some suggestion that it is accomplished by certain cell appendages, including curli, pili/fimbriae and flagella, or *via* two-component and phosphorelay signalling systems^59^. The fact that the Cpx- and Rcs- signalling systems are both activated upon hydrophobic and hydrophilic surface contact, respectively, is consistent with roles in biofilm development^60–62^. However, it has been suggested recently that Rcs-signalling, but not the Cpx-signalling, is activated upon surface contact and involved in promoting initial biofilm formation^63^.

Hence, ambiguity in the literature surrounds the role of Cpx-signalling during initial surface sensing and subsequent biofilm development. The goal of this study was therefore to assess the role of Cpx-signalling in the homoeostasis of biofilms formed by *Yptb*. This is further motivated by earlier observations that Cpx-signalling reduces adhesion of *Yptb* to eukaryotic cells^64^. Indeed, we report that mutant *Yptb* lacking the dual functional CpxA sensor kinase and phosphatase totally aborted biofilm formation on a plastic surface and on the surface of the *Caenorhabditis elegans* nematode. This substantial reduction in biofilm development was caused in part by an excess of active CpxR~P deregulating expression from the *hms* loci. Therefore, Cpx-signalling prevents biofilm formation by restricting EPS production, the core component of *Yersinia* biofilm extracellular matrix material.

## Results

### Intact Cpx-signalling is crucial for the biofilm formation on diverse surfaces

**Fig. 1:**
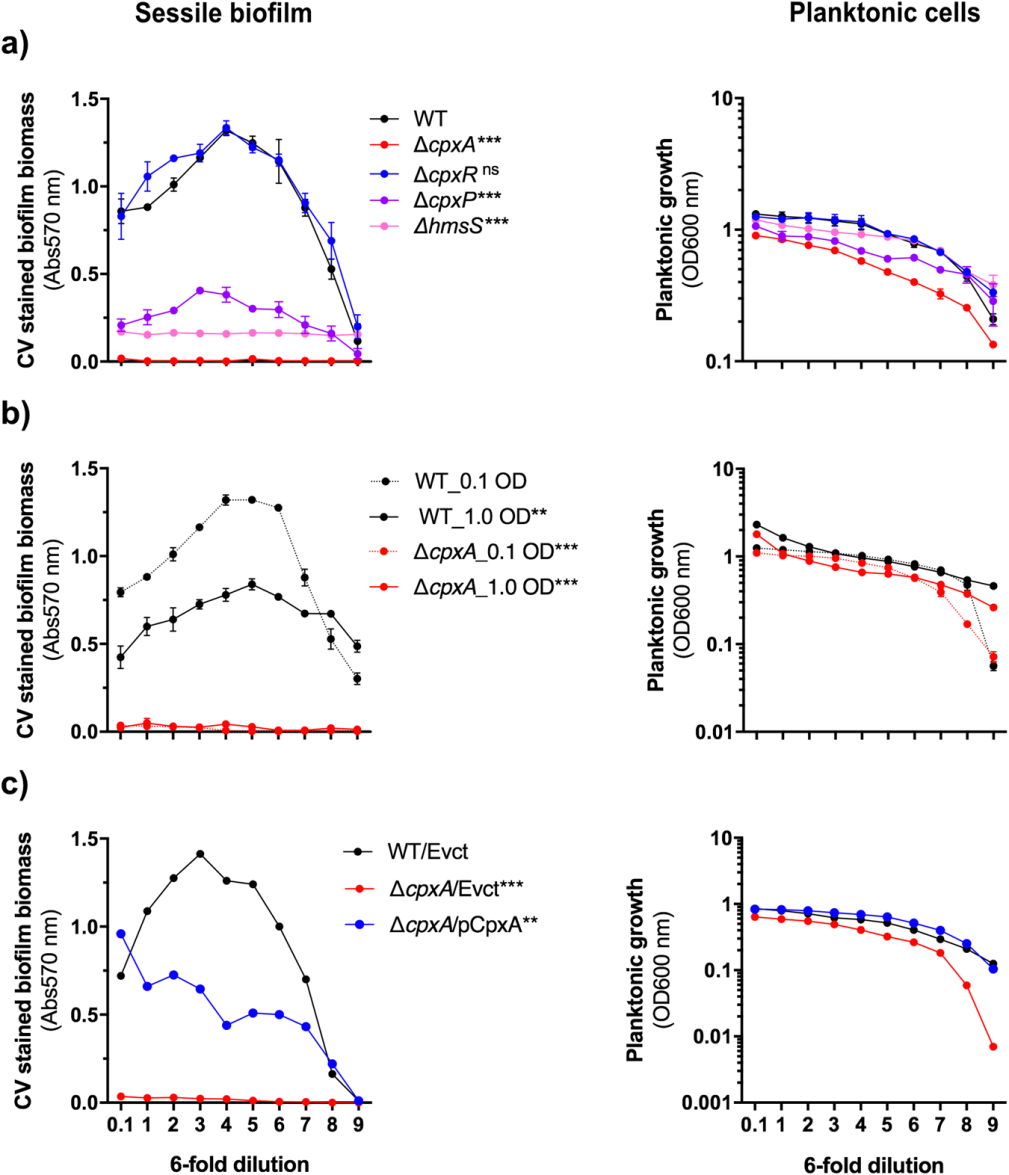
Intact Cpx-signalling is crucial for *Yptb-*YPIII biofilm formation on abiotic surface. Dynamic biofilm formation was analysed in 96-well round-bottom microtiter plates. A serial dilution-based scheme (6-fold, 9 times) was applied to monitor development of sessile biofilm and growth of planktonic cells from the LB-cultured (24 h incubation at 26 °C, 125 rpm shaking) strains of *Yptb-*YPIII. Each dot in the graph represents a different dilution of the same culture. Parental, wild-type (WT) or mutants within the Cpx-signalling were seeded either at 0.1 OD_600_ or both at 0.1 and 1.0 OD_600_. (**a**) Loss of CpxA (Δ*cpxA* null-mutant) prevents biofilm formation. (**b**) Poor growth of the Δ*cpxA* null-mutant does not account for this biofilm formation defect. (**c**) Biofilm formation is restored to the Δ*cpxA* null-mutant *via* ectopic expression of wild-type copy of *cpxA* in Δ*cpxA* null-mutant of the Cpx-signalling. Error bars on the graphs represent standard error of mean from three biological and three technical replicates of each strain. Statistical differences between sessile biofilm formation of each strain uses a total biofilm biomass counts (represented by the area under each curve) in comparison with a respective reference control (WT for **a**, WT_01 OD for **b** and WT/Evct for **c**). Standard error of mean for each strain was calculated from three independent biological experiments containing three technical replicates. Extent of significance from the parent or negative control was determined using One-way ANOVA with Tukey’s multiple comparisons test, with a single pooled variance. The difference in variance with a *p*-value of <0.05 was considered significant. The *p*-values are indicated by <0.001 (***), <0.01 (**) and > 0.05 (ns).

Due to the ambiguity surrounding the role of Cpx-signalling in surface contact and sensing during biofilm formation^63,64^, we examined the role of *Yptb-*YPIII Cpx-signalling in biofilm formation on two different surfaces—one was an abiotic surface and the other a biotic surface. To monitor biofilm development on an abiotic surface, 96-well polystyrene microtiter plates were used. Biofilm biomass dynamic was assessed following the incubation of 6-fold serially diluted cultures of the parental *Yptb-*YPIII serotype O:3, a food-borne clinical isolate, and the Cpx-signalling isogenic mutants, Δ*cpxA* and Δ*cpxR*. Strikingly, loss of CpxA (Δ*cpxA*) prevents biofilm formation (Fig. 1a, left panel). We noted that planktonic growth of the Δ*cpxA* mutant was slightly compromised as measured by optical density measurements at a wavelength of 600 nm (Fig. 1a, right panel). Since differences in cell wall composition could result in different light scattering characteristics, we also tested the actual number of living cells at similar OD_600_ for the different strains. Regardless of the strain tested, similar numbers of viable bacteria were recovered, although the colonies formed by mutants lacking *cpxA* were smaller and exhibited a different pigmentation (Supplementary Fig. 1). Nevertheless, since biofilm is growth dependent, we repeated the assay with a 10-fold increase in initial culture density. Despite an anticipated increase in planktonic growth (Fig. 1b, right panel), this mutant was still unable to form a biofilm (Fig. 1b, left panel). Importantly, the biofilm defect in the Δ*cpxA* mutant was partially restored *via* the ectopic expression of *cpxA* (Δ*cpxA*/pCpxA), supporting the crucial role of CpxA in biofilm formation (Fig. 1c). These results suggest a vital role for Cpx-signalling in the cellular response to surface contact and sensing by *Yptb* during biofilm development.

**Fig. 2:**
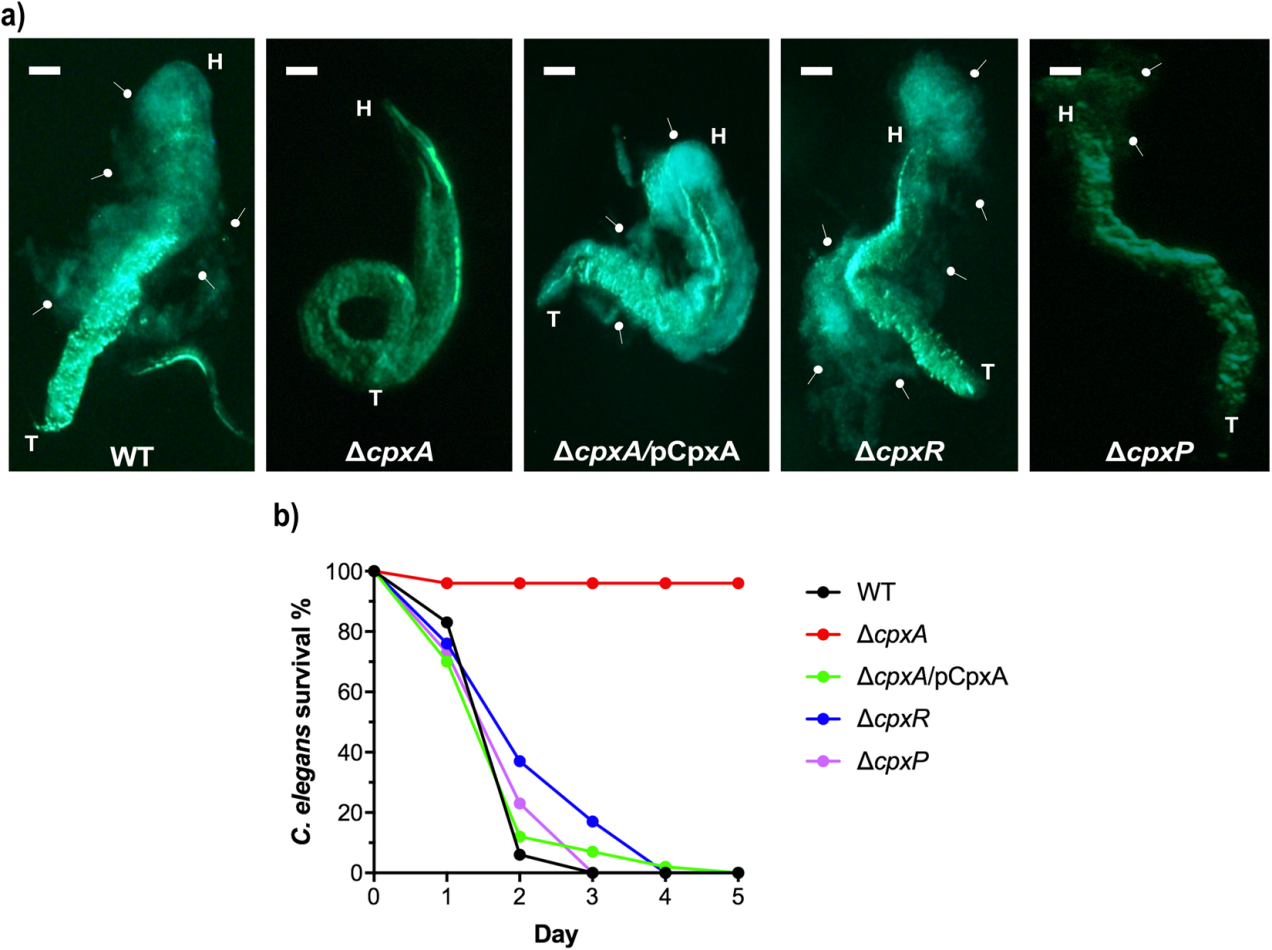
Intact Cpx-signalling is crucial for *Yptb-*YPIII biofilm formation on biotic surface. Infection and survival of N_2,_ a standard laboratory strain of *Caenorhabditis elegans* nematode on the lawn of Cpx-signalling strains. (**a**) L4 stage (larval) nematodes were seeded on the equalised OD_600_ lawn of Gfp*-*expressing parental *Yptb-*YPIII (WT), Δ*cpxA*, Δ*cpxR*, Δ*cpxP* and Δ*cpxA*/pCpxA strains of Cpx-signalling and imaged *C. elegans* after 24 h of growth on each respective lawn. White arrowheads indicate Gfp-coloured dense mass of biofilm from the corresponding strain. The ∆*cpxA* null-mutant failed to form biofilm on the surface of *C. elegans*, which was restored upon ectopic expression of pCpxA. H- head and T- tail of *C. elegans*. A representative image from three independent biological replicates of each strain is shown. Pictures were acquired by Nikon Stereoscopic Microscope SMZ1500 with Gfp-fluorescence (excitation ƛ 470 nm and emission ƛ 525 nm) at ×50 magnification, equivalent to a 10 µm scale bar. The Pre-installed NIS Elements V4.0 imaging software was used to capture the pictures. (**b**) Survivability percentage of *C. elegans* on parental wild-type (WT) and mutant strains of Cpx-signalling. Thirty L4 stage (larval) nematodes were seeded on the equalised OD_600_ lawn of WT, Δ*cpxA*, Δ*cpxA*/pCpxA, Δ*cpxR* and Δ*cpxP* strains and scoring of live nematodes was recorded every 24 h, continuous for 5 days.

To explore further a role for Cpx-signalling in biofilm formation, we utilised the same set of isogenic *Yptb-*YPIII Cpx-signalling variants, but now expressing GFP, to examine their ability to form biofilm on the biotic surface of the *Caenorhabditis elegans* nematode. Larvae at L-4 stage from a standard laboratory strain (N_2_) of *C. elegans* were transferred (*n* = 30) on to normalised lawns of Gfp-expressing *Yptb-*YPIII Cpx-signalling variants. After 24 h growth at room temperature, *C. elegans* were examined by fluorescent microscopy. When exposed to a lawn of parental bacteria or bacteria lacking CpxR, a distinct biofilm biomass was formed on the worm surface (Fig. 2a). In contrast, the surface of worms that had been grown on bacteria lacking CpxA remained completely free of the biofilm biomass (Fig. 2a). However, the Δ*cpxA* mutant strain complemented by ectopic expression of CpxA (Δ*cpxA*/pCpxA) regained the ability to establish a robust biofilm on the surface of exposed *C. elegans* (Fig. 2a). These data corroborate the plastic plate assay, confirming that Cpx-signalling is crucial for *Yptb-*YPIII biofilm development on at least two different surfaces—one being abiotic and the other being biotic. Additionally, we monitored the survival rate of *C. elegans* on the lawns of these strains over a 5-day period. We observed that by the end of day 3, all worms had died on the parental *Yptb-*YPIII (WT) lawn (Fig. 2b). Moreover, growth on the lawns of Δ*cpxR* and Δ*cpxA*/pCpxA caused the death of all worms after 4 days (Fig. 2b). In contrast, all worms survived on the Δ*cpxA* lawn throughout the incubation period (Fig. 2b). Hence, the biofilm biomass formed by WT, Δ*cpxR*, and Δ*cpxA*/pCpxA strains on the worm surface resulted in worm starvation and death. On the other hand, an inability of the Δ*cpxA* mutant to form a biofilm permitted the worms to continue feeding and remain alive. Taking these observations all together, intact Cpx-signalling is clearly crucial for the ability of *Yptb-*YPIII to form biofilms on diverse surfaces.

### Accumulation of active phosphorylated CpxR prevents biofilm formation

**Fig. 3:**
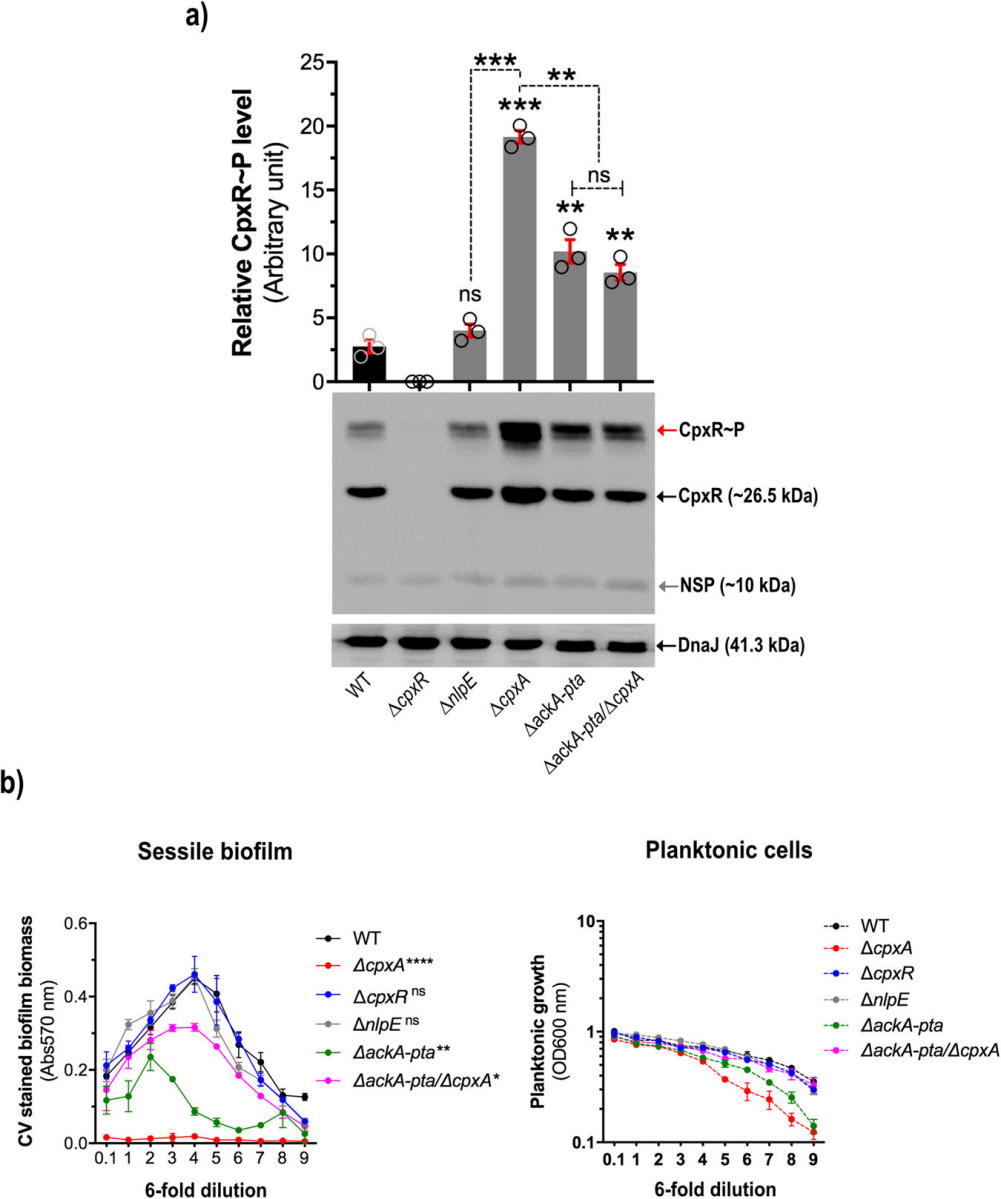
Accumulation of active phosphorylated CpxR prevents biofilm formation. The Phos-tag^TM^ acrylamide system was used to measure accumulated CpxR~P in bacteria grown in LB broth to late-stationary phase at 26 °C in 96-well round-bottomed microtiter plate (**a**). Samples were recovered from the planktonic portion of the culture from the mature stage of biofilm (equivalent to 4^th^ dilution of the 6-fold dilution series). Lysed bacteria were electrophoresed on a freshly prepared 12% Phos-tag^TM^ Acrylamide AAL-107 gel, immunoblotted, and detected with anti-CpxR antiserum. The cytoplasmic molecular chaperone DnaJ, served as a loading control and derived from the same samples, but analysed on a conventional 12% acrylamide SDS-PAGE and immunoblotted with anti-DnaJ antiserum as described in Methods section. The representative unprocessed (raw) blot image of each can be seen in the Supplementary file. Strains: parent (WT), YPIII/pIB102; *cpxR* null-mutant, YPIII08/pIB102; *nlpE* null-mutant, YPIII34/pIB102; *cpxA* null-mutant, YPIII07/pIB102; *ackA, pta* null mutant, YPIII69/pIB102; *ackA, pta* and *cpxA* null-mutant, YPIII49/pIB102. The red arrow reflects the active phosphorylated CpxR~P isoform accumulated in the *Yptb*-YPIII cytoplasm, while the black arrow indicates the accumulated inactive non-phosphorylated CpxR isoform. An unknown non-specific product (NSP) is indicated by a grey arrow. These same strains were monitored for biofilm formation using the serial dilution scheme (6-fold, 9 times) in 96-well round-bottom microtiter plates (**b**). Error bars on the graphs represent standard error of mean from three independent biological and three technical replicates of each strain. Statistical differences between sessile biofilm formation of each strain uses a total biofilm biomass counts (represented by the area under each curve) in comparison with WT as the reference control. Statistical significance with respect to the parent (WT) was determined using One-way ANOVA with Tukey’s multiple comparisons test, with a single pooled variance. The difference in variance with a *p*-value of <0.05 was considered significant. The *p*-values are indicated by <0.001 (***), <0.01 (**) and >0.05 (ns).

In several different bacteria it has been demonstrated that a deletion of *cpxA* leads to an excess of CpxR~P as CpxR is phosphorylated *via* alternate pathways such as through the low molecular weight phosphodonor Acetyl-phosphate^49,65–68^. Hence, we wondered if an excess of active CpxR~P in the *cpxA* strain of *Yptb-*YPIII prevents biofilm formation. Protein lysates were prepared from the biofilm biomass derived from the microtitre plate serial dilution assay. When analysed by the Phos-tag^TM^ acrylamide gel system it was evident that this material contained active phosphorylatable CpxR~P accumulated in the *cpxA* null-mutant (Supplementary Fig. 2). Specificity of this assay was validated by using an isogenic mutant encoding *cpxR*_D8A, D9A, D51A, M53A, K100A_ that produces a non-phosphorylated CpxR (CpxR_Pneg_). This CpxR_Pneg_ variant migrated only as the inactive non-phosphorylated isomer (Supplementary Fig. 2). Thus, to address if this accumulation prevented biofilm formation, we utilised a set of isogenic mutations lacking the *ackA* and *pta* genes responsible for the production of the high-energy phospho intermediate, Acetyl-phosphate—one in the CpxA-plus (WT) background and one in the CpxA-minus (Δ*cpxA*) background. Significantly, the triple mutant lacking *cpxA, ackA* and *pta* did not accumulate CpxR~P to high levels as judged by the Phos-tag^TM^ acrylamide gel system (Fig. 3a). Hence, an inability to produce the high-energy phospho intermediate, Acetyl-phosphate, reduces the amount of active phosphorylatable CpxR~P accumulated in the *cpxA* null-mutant. When these strains were assessed for biofilm formation by microtitre plate serial dilution assay, introduction of the *ackA* and *pta* mutations into the *cpxA* mutant restored the ability to produced biofilms, albeit not to the extent observed for parental (WT) bacteria (Fig. 3b). Thus, this data provides compelling evidence for a role of active phosphorylated CpxR~P in preventing biofilm formation by *Yptb*.

### The *hms* loci of *Yptb*-YPIII are transcriptionally regulated by active Cpx-signalling

**Fig. 4:**
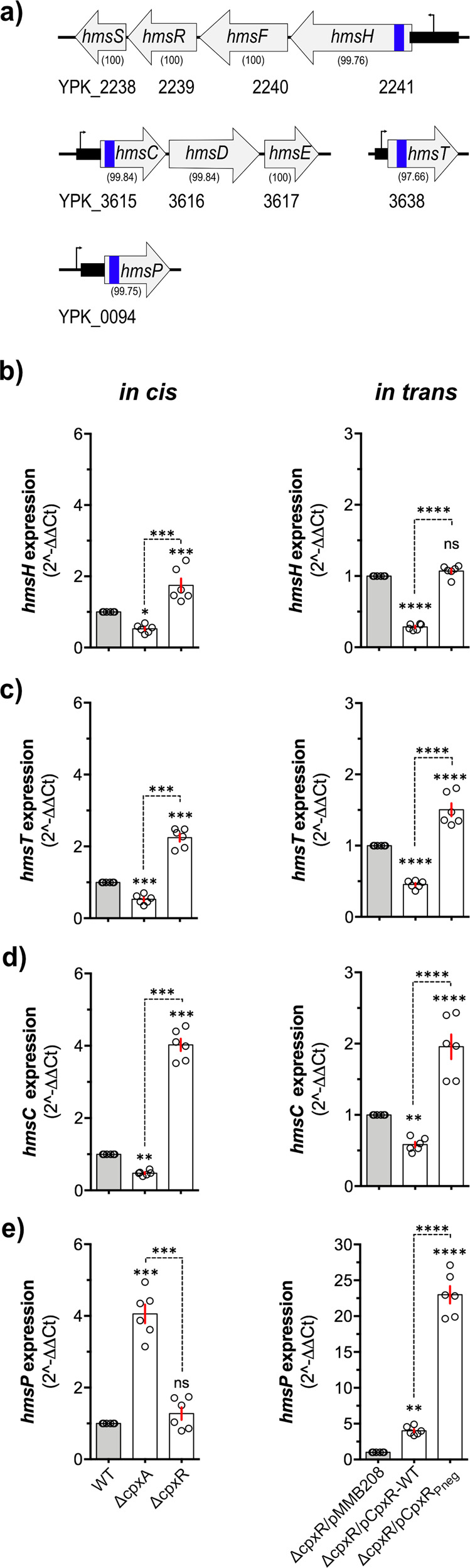
Relative expression of the *hms* loci is influenced by accumulation of active phosphorylated CpxR. (**a**) Operon structure of the *Yptb-*YPIII *hms* loci. Genetic organisation of *hmsHFRS* and *hmsCDE* operons and unlinked *hmsT* and *hmsP* genes. The locus-tag (YPK_XXXX) of each loci mentioned underneath. PCR-amplified gene-specific (for qRT-PCR) and 5' UTR (for EMSA) fragments of corresponding loci are represented by respective vertical blue and horizontal black rectangle box. Numbers in parentheses indicate amino acid sequence identity with isofunctional homologues from *Ype* strain KIM10+. (**b**–**e**) Cpx-signalling mediated differential expression of *hms* loci. Quantitative RT-PCR was performed on the cDNA template, synthesised from the total RNA of *Yptb-*YPIII Cpx-signalling *in cis* strains, parental WT (intact Cpx-signalling), Δ*cpxA* (locked-on Cpx-signalling producing excessive active phosphorylated CpxR~P) and Δ*cpxR* (locked-off Cpx-signalling unable to produce CpxR) and from in trans strains, Δ*cpxR*/pMMB208 (ectopic expression of IPTG-inducible pMMB208 plasmid in Δ*cpxR* null-mutant, negative control), Δ*cpxR*/pCpxR-WT (ectopic expression of wild-type CpxR from pMMB208 plasmid in Δ*cpxR* null-mutant) and Δ*cpxR*/pCpxR_Pneg_ (ectopic expression of phosphorylation defective CpxR_Pneg_ mutant from pMMB208 plasmid in Δ*cpxR* null-mutant). Both *in cis* and in trans strains were cultured in LB with shaking (150 rpm) at 26 °C. The *in cis* strains were grown for 24 h (no IPTG) while the in trans strains were grown up to 6 h with IPTG-induction (10 µM) following subculture (1/20 dilutions). Expression of the *hms* loci within the *in cis* strains, Δ*cpxA* and Δ*cpxR* was calculated relative to WT (**b**–**e**; left panel) whereas expression of corresponding *hms* gene within the in trans strains, Δ*cpxR*/pCpxR-WT and Δ*cpxR*/pCpxR_Pneg_ was calculated relative to Δ*cpxR*/pMMB208 (**b**–**e**; right panel). Relative expression of *hms* loci from both *in cis* and in trans was monitored from three biological and three technical replicates of each strain with two house-keeping internal standards, *gyrB* and *rpoC*. Statistical significance was determined using One-way ANOVA with Tukey’s multiple comparisons test, with a single pooled variance. The difference in variance with a *p*-value of < 0.05 was considered significant. The *p*-values are indicated by <0.0001 (****), <0.001 (***), <0.01 (**), <0.05 (*) and >0.05 (ns).

As demonstrated above, Cpx-signalling is crucial for biofilm formation, irrespective of the surface. We hypothesised that active CpxR~P acts as a transcription factor regulating one or more aspects of biofilm formation. EPS production controlled by the *hms* loci is a vital component of *Yptb* biofilm^9,12^. We could also verify this in our experimental systems. A Hms-EPS defective strain created by deletion of the *hmsS* (∆*hmsS*) that encodes for the biofilm PGA (Poly-β-1, 6-GlcNAc) synthesis protein, PgaD/HmsS was unable to form any measurable biofilm biomass (Fig. 1a), in agreement with previous observations^20^. The lack of biofilm biomass was equivalent to the ∆*cpxA* mutant (Fig. 1a). Hence, we investigated whether the ∆*cpxA* mutant phenotype was caused by active CpxR~P controlling transcription from the *hms* loci. We inspected the transcriptional profile of four *hms* loci—*hmsHFRS, hmsCDE, hmsT* and *hmsP* (Fig. 4a). In the first approach, qRT-PCR was used to measure endogenous transcriptional expression of the *hms* loci located *in cis* in *Yptb* of the locked-on Cpx-signalling mutant, Δ*cpxA* and the locked-off CpxR defective mutant, Δ*cpxR*, in comparison with parent (WT) bacteria. Following late-stationary phase growth, it was observed that active CpxR~P (in Δ*cpxA*) significantly reduced the expression of the Hms-EPS transporter gene, *hmsH* (~50% reduction cf WT) (Fig. 4b), and the diguanylate cyclase encoding genes, *hmsT* (~45% reduction cf WT) (Fig. 4c) and *hmsC* (~50% reduction cf WT) (Fig. 4d), which synthesise c-di-GMP, a vital molecule for the synthesis and export of Hms-EPS on the cell surface^16^. This repressive effect was relieved in Δ*cpxR* null-mutant, confirming the active contribution of CpxR~P as a transcriptional repressor of expression from the *hmsHFRS, hmsT* and *hmsCDE* loci (Fig. 4b–d—in cis). This CpxAR-dependent collective loss of expression from these three loci would be expected to reduce EPS synthesis and transport to the bacterial surface. In contrast, we also observed that active CpxR~P increased the relative expression of *hmsP* (Fig. 4e), which encodes a diguanylate phosphodiesterase that hydrolyses c-di-GMP^19,24^. Critically, a CpxAR-dependent increase of HmsP will decrease c-di-GMP levels, which will also hinder EPS production.

To validate these data, we performed qRT-PCR analysis of *hms* loci within the Δ*cpxR* mutant ectopically producing from an expression plasmid either an active wild-type (pCpxR+) or phosphorylation defective inactive isoform of CpxR (pCpxR_Pneg_). Mirroring the “*in cis*” generated data, accumulation of active CpxR~P by overexpression of CpxR~P resulted in repression of *hmsH, hmsT* and *hmsC* expression (Fig. 4b–d—in trans), and activation of *hmsP* expression (Fig. 4e—in trans). On the other hand, accumulation of inactive non-phosphorylated CpxR_Pneg_ resulted in activation of *hmsH, hmsT* and *hmsC* expression (Fig. 4b–d—in trans). Interestingly, this phosphorylation defective CpxR_Pneg_ also lead to robust de-repression of *hmsP* (Fig. 4e—in trans). This indicates that the CpxR-HmsP intrinsic negative feedback loop might be eliminated by ectopic expression of an IPTG-inducible form of inactive CpxR.

In parallel, we also established translational gene fusions of *hmsH, hmsT* and *hmsP* with *gfpmut3* in the backgrounds of parent *Yptb*-YPIII (WT), Δ*cpxA* mutant and Δ*cpxR* mutant. Fluorescence intensity output from the P_*hmsH*_::Gfp (Supplementary Fig. 3a) and P_*hmsT*_::Gfp (Supplementary Fig. 3b) fusions were considerably lower in the Δ*cpxA* null-mutant compared to the Δ*cpxR* null-mutant. Moreover, fluorescence intensity output from the P_*hmsP*_*::*Gfp fusion was considerably higher in the Δ*cpxA* null-mutant compared to the Δ*cpxR* null-mutant (Supplementary Fig. 3c). These data corroborated observations from qRT-PCR experiments. Taken altogether, we show clearly that active CpxR~P represses transcriptional output from the *hmsHFRS, hmsT* and *hmsCDE* loci, but induces output from the *hmsP* locus. This differential control is explained by the opposing roles these loci have in EPS synthesis in *Yptb*-YPIII.

### CpxR~P-dependent control of *hms* transcription is direct

**Fig. 5:**
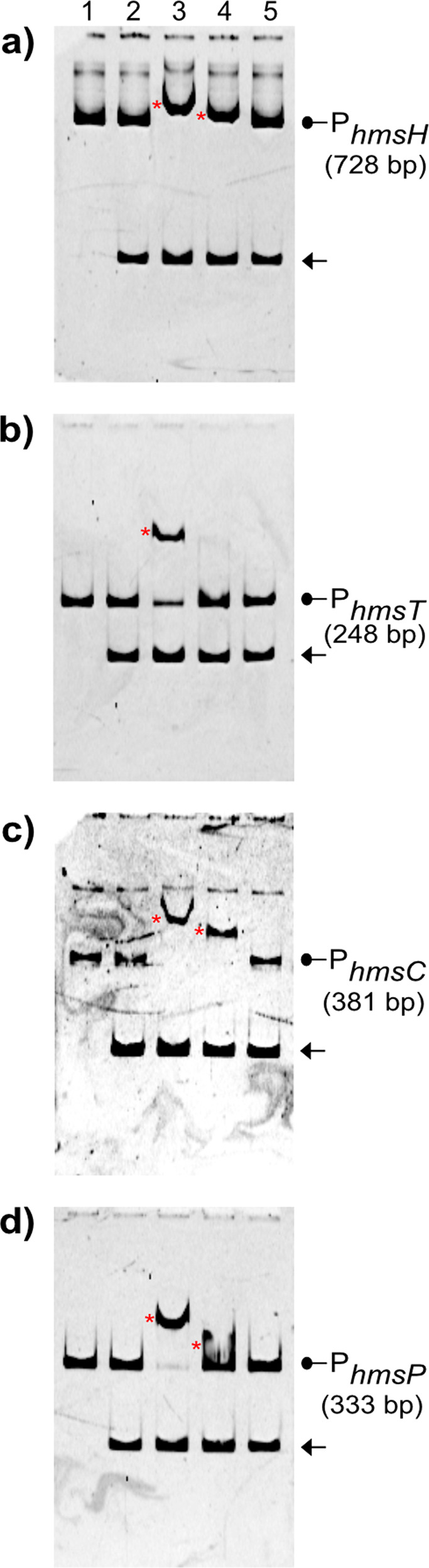
Active phosphorylated CpxR~P binds at the promoter of *hms* loci. An EMSA with complete 5' intergenic regulatory DNA of indicated *hms* loci with active CpxR~P was used to measure specific protein-nucleic acid interactions. Red asterisks (*) indicate the target promoter DNA-CpxR_wt_ complex. Unbound promoter DNA is indicated with an arrowhead. The inactive non-phosphorylatable CpxR_Pneg_ isoform was unable to bind target DNA at these same conditions. A 16S rDNA fragment (148 bp) used as ‘non-specific’ negative control and its running location is indicated by an arrow (←). Lane-1: target promoter DNA, Lane-2: target promoter plus non-specific 16S rDNA, Lane-3: target promoter, non-specific 16S rDNA and CpxR_His6_ (100 µM), Lane-4: target promoter, non-specific 16S rDNA and CpxR_His6_ (50 µM) and Lane-5: target promoter, non-specific 16S rDNA and phosphorylation defective Mt7-CpxR_His6_ (100 µM). Each reaction contained 200 mM Acetyl-phosphate to phosphorylate CpxR_His6_ in the respective lane. Gel were stained with 1× GelRed DNA-staining dye solution. A representative image from three independent experiments for each target is shown. The representative unprocessed (raw) image of each EMSA-gel can be seen in the Supplementary file.

Having established that active CpxR~P represses transcriptional output from the *hmsHFRS, hmsT* and *hmsCDE* loci, but induces output from the *hmsP* locus, we wondered if this regulatory control was direct. Hence, we examined the in vitro binding of an active phosphorylated form of purified CpxR (CpxR_wt_) and an inactive non-phosphorylated form of purified CpxR (CpxR_Pneg_) at the promoter regions of the four *hms* loci, P_*hmsH*_, P_*hmsT*_, P_*hmsC*_ and P_*hmsP*_ as depicted in Fig. 4a. Two concentrations of purified CpxR_His6_ −50 µM and 100 µM, were tested for specific binding. In all cases, the higher concentration of active phosphorylated CpxR_wt_ caused a shift of all four specific *hms* DNA fragments (Fig. 5). This was specific targeted binding because inactive non-phosphorylated CpxR_Pneg_ failed to induce any shift in the four specific *hms* DNA fragments under identical conditions, and neither CpxR_wt_ nor CpxR_Pneg_ induced a shift of the 16 S rRNA encoding DNA fragment that was used as another specificity control (Fig. 5). Hence, phosphorylated CpxR~P is required to bind to the promoters of the four *hms* loci. Further, this direct binding is likely a major mechanism driving CpxR~P dependent repression of *hmsHFRS, hmsT* and *hmsCDE* transcription, and induction of *hmsP* transcription.

### Cpx-signalling dependent transcriptional repression of the *hms* loci reduces EPS production

**Fig. 6:**
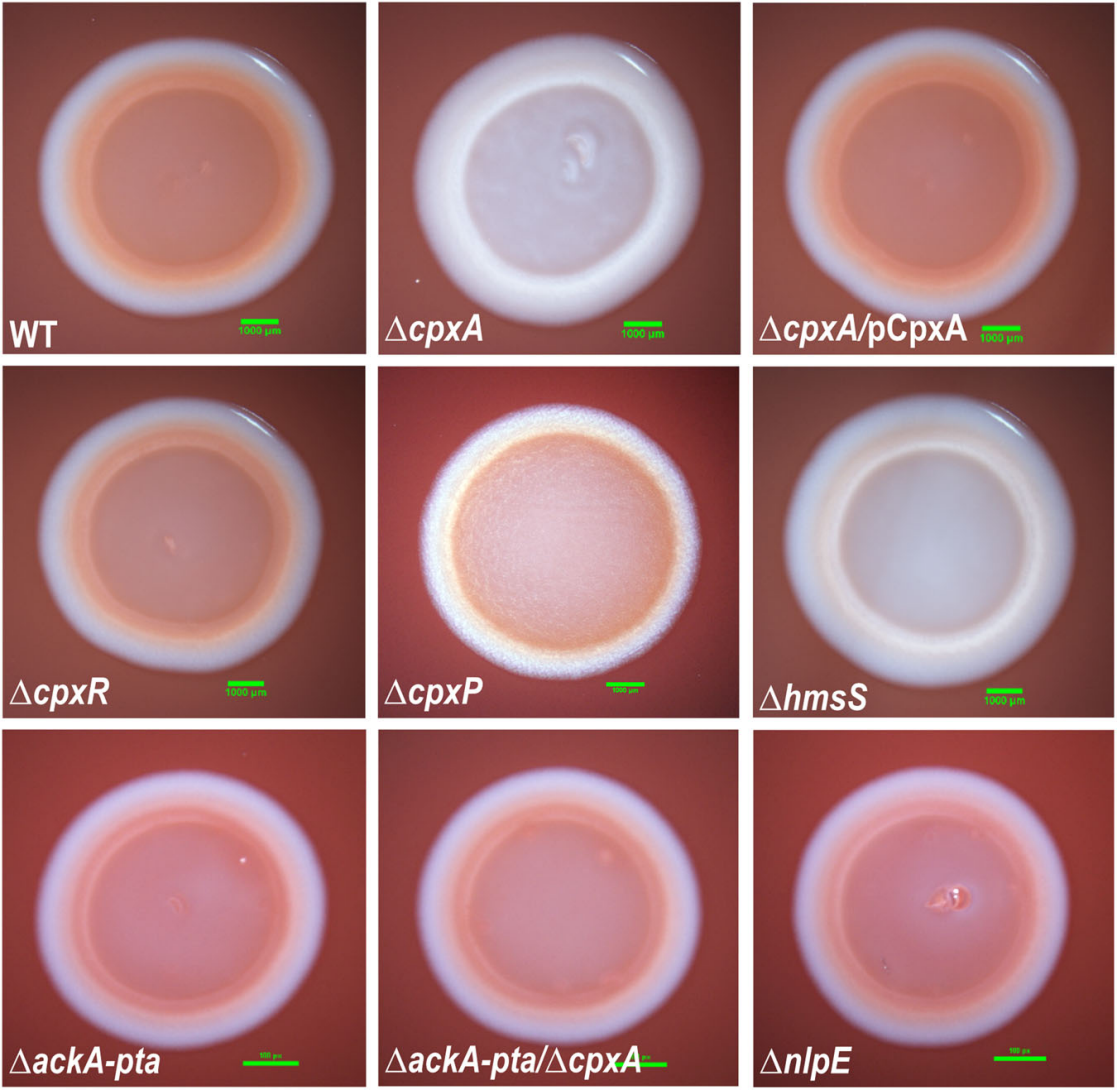
Hms-dependent production of EPS is suppressed by in vivo accumulation of active CpxR~P. The Congo Red binding assay was used as an indicator of EPS production by various *Yptb*-YPIII strains. Indicated *Yptb* strains were grown (with shaking) at 26 °C for 18 h in LB broth lacking NaCl. A 3.5 µL culture containing an equal number of cells via normalisation of optical density at 600 nm was spotted on NaCl-lacking LA that was supplemented with the dye 0.01% (w/v) Congo Red. Images were taken after growth at 26 °C for 24 h. A representative colony from three independent biological and three technical triplicates of each strain is shown. Used as a negative control was the ∆*hmsS* mutant that contains an in-frame deletion of codons 11–135 in *hmsS*, a crucial gene for the Hms-EPS matrix synthesis and export. Indicated scale bar (green line) is 1000 µm. Strains: parent (WT), YPIII/pIB102; *cpxA* null-mutant, YPIII07/pIB102; complemented *cpxA*/pCpxA^+^, YPIII07/pIB102, pJF067; *cpxR* null-mutant, YPIII08/pIB102; *cpxP* null-mutant, YPIII41/pIB102; *hmsS* null- mutant, YPIII_2238/pIB102; *ackA, pta* null-mutant, YPIII69/pIB102; *ackA, pta* and *cpxA* null-mutant, YPIII49/pIB102; *nlpE* null-mutant, YPIII34/pIB102.

Having established that active CpxR~P differentially regulates transcription of the *hms* loci, we investigated if this correlated with altered Hms-EPS synthesis and export—an essential component of a cohesive *Yersinia* biofilm^16^. The *Yersinia-*Congo Red-binding assay is a traditional way to determine whether *Yersinia* strains manufacture and export Hms-EPS on the cell surface^18,20,37^. Colonies of the locked-on CpxA defective strain (∆*cpxA*) were unable to bind Congo Red (Fig. 6). This mirrored the phenotype of colonies of the Hms-EPS defective strain, ∆*hmsS*, which were also unable to bind Congo Red (Fig. 6). Crucially, colonies of parental *Yptb*-YPIII (WT), the ∆*cpxR* and ∆*cpxP* null-mutants, and the complemented Δ*cpxA*/pCpxA^+^ strain were all found to adsorb the Congo Red dye giving a distinctive red appearance (Fig. 6). Moreover, the *cpxA* null-mutant that also lacked the *ackA* and *pta* genes, regained the ability to bind Congo Red (Fig. 6). This emphasises that it is the accumulation of active phosphorylated CpxR~P that limits the ability of *Yptb* to bind Congo Red. Taken together, these results signify that *Yptb-*YPIII Cpx-signalling differentially regulates *hms* loci transcription, and this combined repression of *hmsHFRS, hmsT* and *hmsCDE* transcription, and induction of *hmsP* transcription prevents the manufacture and export of EPS to the cell surface. Hence, the lack of EPS represents one major reason why the *Yptb*-YPIII Δ*cpxA* null*-*mutant is unable to develop a biofilm.

### Contribution of auxiliary Cpx-signalling molecules, CpxP and NlpE, on biofilm formation

CpxP and NlpE are auxiliary proteins involved in environmental signal integration in the Cpx-signalling pathway. The periplasmic located CpxP inhibits the histidine kinase ‘sensor’ function of CpxA through a direct dynamic interaction^69,70^, whereas the outer membrane located NlpE contributes to system activation^60,62^. To address if CpxP and NlpE of *Yptb*-YPIII contribute to biofilm formation we examined the phenotype of a full-length in-frame *cpxP* mutant and a full-length in-frame *nlpE* mutant in our biofilm assays. The *cpxP* mutant formed biofilm on abiotic surface as judged by the plastic microtitre plate serial dilution assay, albeit to a reduced extent compared to parental *Yptb* (Fig. 1a). On the other hand, robust biofilms were also formed on the biotic surface according to the *C. elegans* grazing assay (Fig. 2a). Critically, the biofilms formed by the *cpxP* null-mutant on the worm body quickly resulted in worm starvation as was similarly observed for worms exposed to wild-type bacteria (Fig. 2b). Moreover, the *cpxP* mutant retained the ability to produce EPS as indicated by colonies stained with Congo red (Fig. 6). The ability of the *cpxP* mutant to form biofilms and maintain EPS production correlates with this bacteria accumulating very little active phosphorylated form of CpxR (Supplementary Fig. 2). Additionally, the *nlpE* null-mutant formed considerable biofilm on the abiotic surface that could not be distinguished from the biofilms formed by parental bacteria (Fig. 3b). Expectantly, the *nlpE* null-mutant retained the ability to produce EPS as judged by colonies stained with Congo Red (Fig. 6). Furthermore, the ability of the *nlpE* null-mutant to form biofilms and maintain EPS production correlates with this bacteria accumulating very little active phosphorylated form of CpxR (Fig. 3a). Hence, the mechanism by which Cpx-signalling regulates biofilms formed by *Yptb* to a minor extent involves CpxP, but is independent of NlpE.

### CpxAR can act through RpoE signalling

**Fig. 7:**
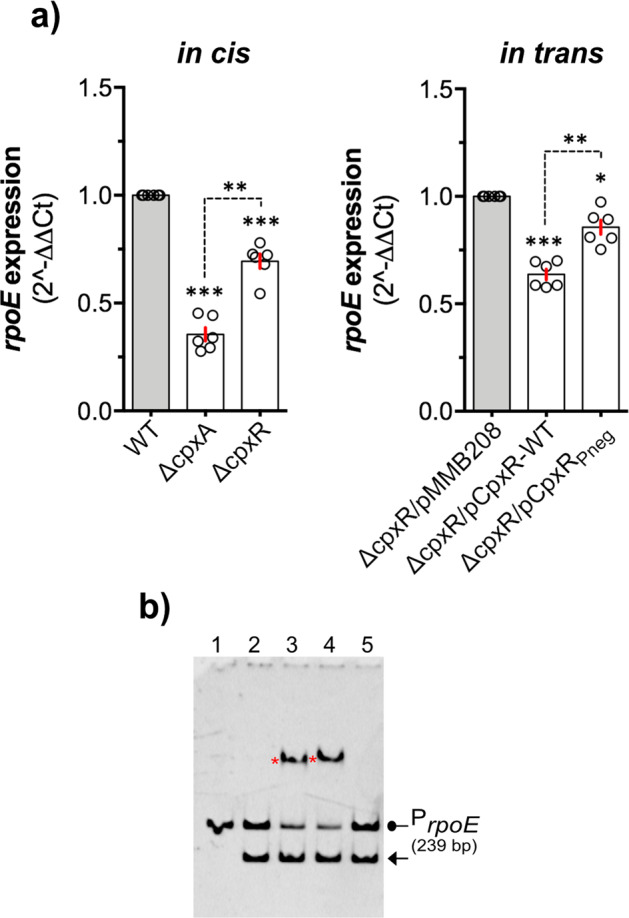
Cpx-signalling mediates transcriptional regulation of *rpoE*. Cpx-signalling mediated repression of *rpoE* transcription (**a**). Quantitative RT-PCR was performed on the cDNA template, synthesised from the total RNA isolated from the *Yptb*-YPIII isogenic ‘*in cis*’ mutant strains: parental WT (intact Cpx-signalling), Δ*cpxA* (locked-on Cpx-signalling with excessive accumulation of active phosphorylated CpxR~P) and Δ*cpxR* (locked-off Cpx-signalling unable to produce CpxR), and from ‘in trans’ strains: Δ*cpxR*/pMMB208 (ectopic expression of IPTG-inducible pMMB208 plasmid in Δ*cpxR* null mutant—negative control), Δ*cpxR*/pCpxR_WT_ (ectopic expression of wild-type CpxR from pMMB208 plasmid in Δ*cpxR* null mutant) and Δ*cpxR*/pCpxR_Pneg_ (ectopic expression of phosphorylation defective CpxR_Pneg_ mutant from pMMB208 plasmid in Δ*cpxR* null mutant). Both *in cis* and in trans strains were cultured in LB with shaking (150 rpm) at 26 °C. The *in cis* strains were grown for 24 h (no IPTG) while the in trans strains were grown up to 6 h with IPTG-induction (10 µM) following subculture (1/20 dilutions). Expression of *rpoE* within the *in cis* strains, Δ*cpxA* and Δ*cpxR* was calculated relative to WT (left panel) whereas expression within the in trans strains, Δ*cpxR*/pCpxR_WT_ and Δ*cpxR*/pCpxR_Pneg_ was calculated relative to Δ*cpxR*/pMMB208 (right panel). Relative expression of *rpoE* from both *in cis* and in trans was monitored from three independent biological and three technical replicates of each strain with two house-keeping internal standards, *gyrB* and *rpoC*. Statistical significance was determined using One-way ANOVA with Tukey’s multiple comparisons test, with a single pooled variance. The difference in variance with a *p*-value of <0.05 was considered significant. The *p*-values are indicated by, <0.001 (***), <0.01 (**) and <0.05 (*). The active phosphorylated form of CpxR (CpxR~P) binds at the promoter of *rpoE* (**b**). EMSA with complete 5' intergenic regulatory DNA of *rpoE* with active CpxR~P. A single asterisk (*) indicates the target promoter DNA-CpxR_wt_ complex. Unbound promoter DNA is indicated with an arrowhead. A 16S rDNA fragment (148 bp) used as ‘non-specific’ negative control and its running location is indicated by an arrow (←). Inactive non-phosphorylated CpxR (CpxR_Pneg_) was unable to bind target DNA. Lane-1: target promoter DNA, Lane-2: target promoter plus non-specific 16S rDNA, Lane-3: target promoter, non-specific 16S rDNA and CpxR_His6_ (100 µM), Lane-4: target promoter, non-specific 16S rDNA and CpxR_wt_ (50 µM) and Lane-5: target promoter, non-specific 16S rDNA and phosphorylation defective CpxR_Pneg_ (100 µM). Each reaction contained 200 mM Acetyl phosphate to phosphorylate CpxR_wt_ in the respective lane. Gel were stained with 1× GelRed DNA-staining dye solution. A representative image from three independent experiments is shown. The representative unprocessed (raw) image of EMSA-gel can be seen in the Supplementary file.

In some bacteria, alternative sigma factor, RpoE contributes to biofilm formation^71–73^. It is not known if this is true also for *Yersinia* spp. because a growth dependence on RpoE makes deficient mutants difficult to study^74–76^. Nevertheless, CpxAR has been shown to act through RpoE in several bacteria^66,77–79^. Hence, here we aimed to determine the impact of *cpxA* and *cpxR* deletions on *rpoE* transcription in *Yptb*-YPIII, with a view that this might be a mechanism through which Cpx-signalling can act. We performed qRT-PCR to measure differential transcriptional expression of the *rpoE* gene in the intrinsically active Cpx-signalling mutant, Δ*cpxA*, and the CpxR defective mutant, Δ*cpxR*, in comparison with parent (WT) *Yptb*, following late-stationary phase growth. It was observed that active CpxR~P (in Δ*cpxA*) significantly reduced the expression of *rpoE* (~50% reduction cf WT) (Fig. 7a). This repressive effect was relieved in the Δ*cpxR* null-mutant, indicating an active contribution of CpxR~P as a transcriptional repressor of expression from *rpoE* (Fig. 7a). Consistent with this, active phosphorylated form of purified CpxR (CpxR_wt_) bound to the *rpoE* promoter region in vitro, whereas the inactive non-phosphorylated form of purified CpxR (CpxR_Pneg_) did not (Fig. 7b). Correspondingly, we identified a putative CpxR~P binding motif reminiscent of the reported consensus sequence GTAAA-N(4-8)-GTAAA^78,80^, which may represent the CpxR binding site within the *rpoE* promoter (Supplementary Fig. 4). Taken together, this data establishes that CpxAR can act through RpoE signalling, which indicates that this Cpx-RpoE regulatory cascade may influence biofilms formed by *Yptb*.

## Discussion

In this study, we established that Cpx-signalling is vital for the initiation, maturation and maintenance of biofilms formed by *Yptb*. This connection remained intact irrespective of using an abiotic or biotic surface for the development of biofilm. Although *Yersinia* spp. can generate different biofilm matrices, the Hms-EPS—Poly-β-1,6-GlcNAc—is a predominant factor of biofilms formed by *Yersinia* spp.^11,16^. Controlled Hms-EPS production and assembly requires four distinct loci; *hmsHFRS, hmsT, hmsCDE* and *hmsP*. The *hmsHFRS* operon encodes for four membrane components that are essential for EPS biosynthesis and export^14,16,18,21^. The *hmsD* and *hmsT* genes encode for two diguanylate cyclase enzymes required for the production of c-di-GMP^22,23^. The c-di-GMP is a second messenger molecule critical for *Yersinia* biofilm production^22^. On the other hand, *hmsP* encodes a phosphodiesterase that acts as a counter balance, enabling *Yersinia* spp. to hydrolyse c-di-GMP to restrict biofilm formation^19,24^. We could demonstrate that active CpxR~P isoform directly targets promoters of the *hmsHFRS, hmsT* and *hmsCDE* operons causing down-regulation of gene expression. Active CpxR~P isoform also directly targets the promoter of *hmsP* causing up-regulation of gene expression. The net effect of this CpxR-mediated differential repression and activation is reduced synthesis and export of Hms-EPS to the cell surface, which severely restricts biofilm development. This suggests that *Yptb* utilises the CpxA-CpxR two-component signalling pathway for surface contact and sensing, which is consistent with initial evidence of a similar role in *E. coli*^60^. These findings add to the many examples that demonstrate the ability of CpxR~P to act as a transcriptional repressor and as a transcriptional activator in diverse bacteria^53–56^.

Biofilm development is a multifactorial process involving a large number of structural components, metabolic processes and regulatory circuitry. This enables bacteria to transition through intial bacterial attachment to a surface, microcolony formation, biofilm maturation, and dispersal to permit bacteria to establish new biofilms in favourable conditions elsewhere. These processes require a number of surface associated factors including pili/fimbriae, flagella, other adhesive fibers as well as carbohydrate-binding proteins^81–83^. This is true also for *Yersinia* biofilm formation, which involves a number of structural factors in addition to Hms-EPS production^11,25,27^. Hence, future work will need to investigate the possibility that Cpx-signalling influences the expression of biofilm structural components other than Hms-EPS.

**Fig. 8:**
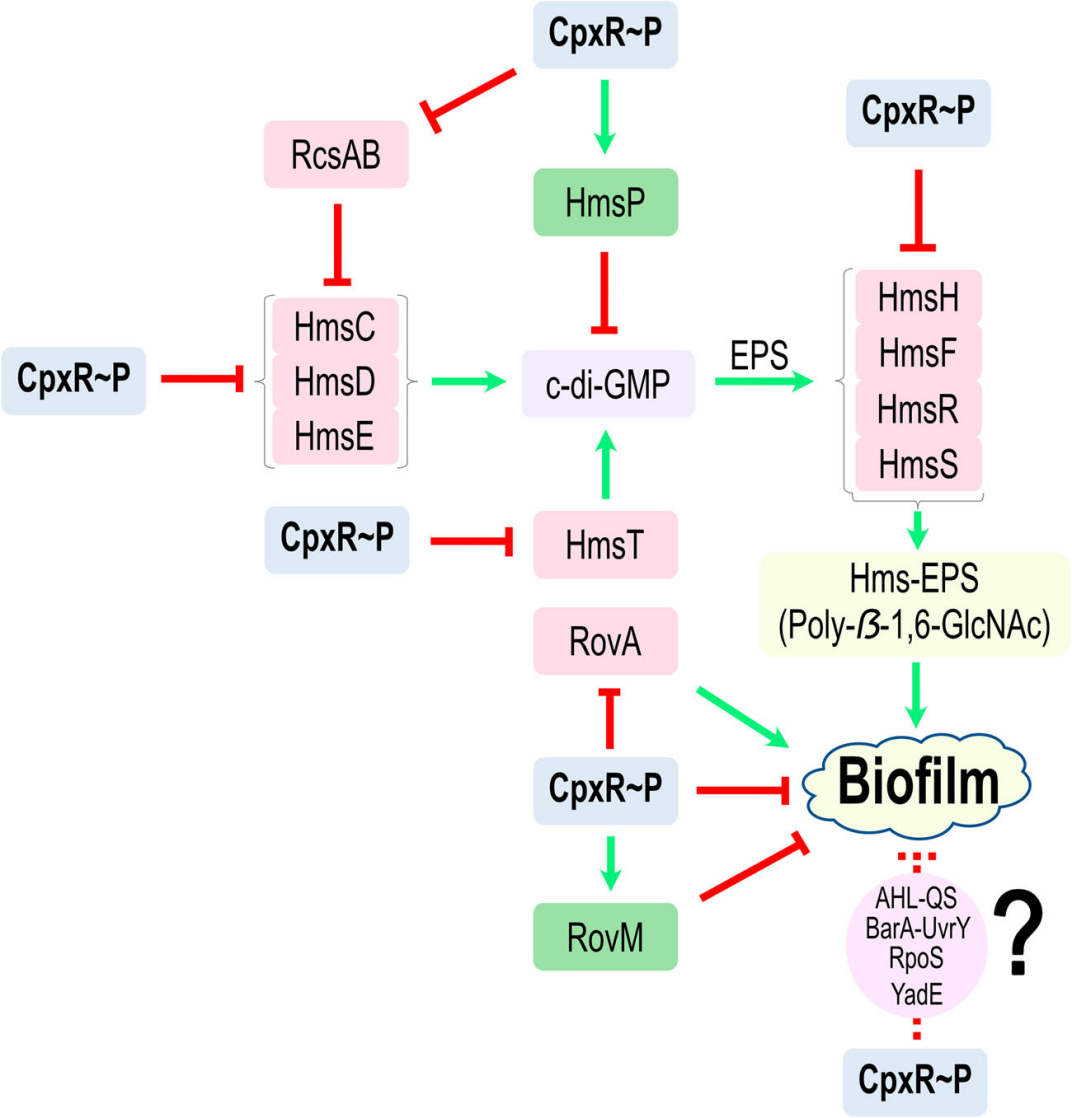
A scheme depicting CpxR~P influence on the *hms* loci expression in *Yptb-*YPIII. A regulatory cascade involving the second messenger signalling molecule, c-di-GMP, is a vital component for the synthesis of Hms-EPS on the cell surface. The *hmsCDE* operon and *hmsT* gene encode diguanylate cyclase (synthesise c-di-GMP), which then helps in the biosynthesis and export of EPS on the cell surface by the *hmsHFRS* operon. The level of c-di-GMP is controlled by the product of *hmsP*, which encodes phosphodiesterase and assists in the hydrolysis of c-di-GMP. Active CpxR~P influences expression of the *hms* loci, activating *hmsP* (represented by green arrow) and repressing both *hmsCDE* and *hmsHFRS* operons along with *hmsT* gene (indicated by red hammer). For clarity, other regulatory cascades controlling Hms-EPS and subsequent biofilm formation are not shown. As biofilm is complex, incorporating many regulatory cascades, these regulatory cascades controlling biofilm (dotted line) could work through Cpx-signalling. These potential connections were not the focus of this study; they remain to be investigated, and this fact is indicated by the question mark ‘?’ symbol.

Similarly, we expect that regulators and signalling cascades other than Cpx-signalling would also work to mediate the development, maintenance and renewal of *Yptb* biofilms. As illustrated in our model (Fig. 8), the involvement of other regulatory elements in controlling biofilm development has already been established using *Yptb*. RovA-RovM is a global regulatory system that likely influences multiple aspects of biofilm development, such as motility and EPS production^30^. The RcsA-RcsB phosphorelay system moderates EPS production by targeting the *hmsT* and *hmsCDE* operons^32^, and also influences biofilm stability through control of the YadE protein^84^. Moreover, the BarA/UvrY two-component system also impacts on biofilm production and stability, possibly through cascade regulation of CsrB and RcsB^37^. In addition, RpoS can influence biofilm formation via effects on motility through control of *flhDC* gene expression, and by regulating EPS synthesis^36^. Furthermore, N-Acyl homoserine lactone-mediated quorum sensing influences biofilm formation at least in part *via* repression of the plasmid encoded Ysc-Yop type III secretion system^29^. Follow-up work will need to establish whether these pathways work independently or through Cpx-signalling. We have already established existence of a CpxR-RovM-RovA regulatory cascade for the control of *Yptb* adhesion^49,50^ and a CpxR-RcsA-RcsB regulatory cascade for the control of *Yptb* Ysc-Yop type III secretion^51^. Hence, it could be that these regulatory cascades may extend the influence of Cpx-signalling to fine-tuning biofilm development.

In this study, *C. elegans* nematodes were used as a model biotic surface for biofilm development by *Yptb*. We noted over the course of several independent experiments that *Yptb* parental bacteria, the Δ*cpxR* mutant and the Δ*cpxP*, which developed robust biofilms on the nematode surface, and resulting in nematode death, appeared to render alterations in nematode surface appearance, including partial disintegration of the worm. These observations were not observed with *C. elegans* exposed to the ∆*cpxA* mutant. These symptoms could well be associated with an invasive *Yptb* infection with pathological consequences. This is not without precendent for there are studies reporting on *Yersinia* colonisation of the nematode gastrointestinal tract^85,86^. This is interesting because the symptoms we observed are analogous to an acute systemic infection. It is tempting to speculate that these symptoms could relate to the production of factors that would promote biofilm-independent invasion such as the degradation activity of NghA, a β-N-acetylglucosaminidase^17^, or the toxigenic activity of the CNF_Y_ toxin^87^. The *Yptb* genome also enodes a potential chitinase, ChiC (YPK_0693). Hence, our follow-up work intends to examine the existence of biofilm-independent *Yptb* killing of infected nematodes.

We used the food-borne clinical isolate *Yptb*-YPIII serotype O:3^88,89^ as a model system for these studies. On the basis that conserved Cpx-signalling has been verified experimentally in other *Yptb* isolates^51,90^, and also in two other *Yersinia* species—*Y. pestis*^91,92^ and *Y. enterocolitica*^79,93,94^, we assume that Cpx-signalling controls biofilm development in other *Yersinia*. Follow-up experimental work with other *Yersinia* isolates will attempt to verify this predicted connection. A comparison of protein sequence derived from *Yptb*-YPIII and *Y. pestis* (*Ype*-KIM10+) revealed a fully intact CpxAR system in *Ype*, including the periplasmic auxiliary signalling molecules, CpxP and NlpE (Supplementary Fig. 5). The Hms components were also represented in both bacteria (Fig. 4a). Moreover, the intergenic regulatory region between the divergent *cpxP* and *cpxRA* operons (Supplementary Fig. 5), as well as upstream of *nlpE* (Supplementary Fig. 5), *hmsHFRS* (Supplementary Fig. 6), *hmsT* (Supplementary Fig. 7), *hmsCDE* (Supplementary Fig. 8) and *hmsP* (Supplementary Fig. 9) all display high degrees of sequence conservation between *Yptb* and *Ype*. Significantly, these intergenic regions contained identifiable CpxR~P binding motifs reminiscent of the reported consensus sequence GTAAA-N(4-8)-GTAAA^78,80^. Hence, it seems probable that *Ype* derived sequence with minimal nucleotide differences would also support the in vitro binding by active CpxR~P. Follow-up work will strive to confirm this with a suite of experiments designed to explore a conserved role of Cpx-signalling in control of Hms-dependent EPS production in both *Yptb* and *Ype*.

## Materials and methods

### Bacterial strains, plasmids and growth conditions

Bacterial strains and plasmids used in this study are listed in Supplementary Table 1. Unless otherwise specified, bacteria were routinely grown in Luria-Bertani agar or Lysogeny broth^95^ at 26 ˚C with aeration using a shaking incubator. As appropriate, antibiotics—Ampicillin (100 μg/mL), Chloramphenicol (25 μg/mL) or Kanamycin (50 μg/mL) were added to the media/broth.

### Mutants construction

The ∆*hmsS*, ∆*cpxP* and ∆*nlpE* null-mutants were constructed by overlap PCR technique^96^ using the relevant primer combinations listed in Supplementary Table 2. The PCR-amplified fragments were cloned into pJET1.2/Blunt plasmid (Thermo Scientific), and the mutated alleles of *hmsS* (representing a deletion of codons 11–135)*, cpxP* (15–151), and *nlpE* (12–213) were confirmed by sequencing (Eurofins MWG Operon, Ebersberg, Germany) with pJET1.2F and pJET1.2R primers (Supplementary Table 2). The confirmed fragments were subcloned into the suicide plasmid, pDM4, following *Xba*I-*Xho*I restriction enzyme digestion. The ligated plasmids were transformed and maintained in *E. coli* SY327λ*pir*. Plasmids with correct insert were sequenced using the gene-specific ‘A’ and ‘D’ primers, respectively (Supplementary Table 2). Confirmed plasmids were transformed into *E. coli* S17-1λ*pir*, which served as the donor in conjugal matings with parental *Yptb*-YPIII. Mutated alleles were introduced into the *Yptb*-YPIII genome by a double cross-over homologous recombination event, and the ∆*hmsS*, ∆*cpxP* and ∆*nlpE* genotypes were recovered by *sacB*-dependent sucrose sensitivity^97^. Presence of these mutations in the genome of *Yptb* was confirmed by PCR using the gene-specific ‘A’ and ‘D’ primer pair, respectively (Supplementary Table 2) combined with subsequent sequence analysis of the amplified fragment with the same primer pair.

A mutated *cpxR* (YPK_4132) gene containing the site specific mutations—D8A, D9A, D51A, M53A, K100A—was synthetically generated by GenScript Biotech (Piscataway, New Jersey, USA) and contained within the plasmid pDK1011 (Supplementary Table 1). The synthetic DNA fragment contained the full length *cpxR* sequence as well as flanking DNA that was 215 bp upstream and 106 bp downstream of *cpxR*. This DNA fragment of 1032 bp was excised from pDK1011 by restriction digestion with *Xho*I/*Xba*I, and then cloned into the *Xho*I/*Xba*I restricted pDM4, giving rise to the mutagenesis vector pDM-DK1011 plasmid (Supplementary Table 1). The inserted sequence in pDM-DK1011 was confirmed by sequencing using the forward and reverse primers, CatR2 and R6KR, respectively (Supplementary Table 2). Generation of the in cis *cpxR*_D8A, D9A, D51A, M53A, K100A_ mutant encoding a non-phoshorylated CpxR_Pneg_ variant occurred via allelic exchange to reconstitute the ∆*cpxR* mutant (YPIII08/pIB102) as described for the ∆*hmsS*, ∆*cpxP* and ∆*nlpE* mutants. The reconstituted CpxR_Pneg_-producing mutant—YPIII_4132pneg/pIB102—was confirmed by colony-PCR using DK1011-12A and DK1011-12D primers (Supplementary Table 2) and subsequent sequencing of the amplified fragment with two additional primers, DK1011-12B and DK1011-12C (Supplementary Table 2).

### Gfp-translational reporter construction

Promoter fragments, P_*hmsH*_ (708 bp) P_*hmsT*_ (315 bp) and P_*hmsP*_ (378 bp), were PCR-amplified from *Yptb*-YPIII genomic DNA, using corresponding primer pairs (Supplementary Table 2). Respective PCR-fragments were subcloned into a commercial shuttle vector, pJET1.2/blunt (Thermo Scientific) and sequenced using pJET1.2F and pJET1.2R sequencing primers (Supplementary Table 2). Sequence-confirmed fragments were lifted from the shuttle vector by *Sac*I-*Sph*I restriction enzyme digestion, subsequently cloned into *Sac*I and *Sph*I restricted destination plasmid, pNQ705-1, which was then transformed into the *E. coli* SY327λ*pir*. Each promoter fusion, in-frame with Gfp of pNQ705-1 was confirmed by sequence analysis using the CatR2 and GfpR2 primers pair (Supplementary Table 2). The sequence-confirmed Gfp-reporter plasmids were transformed into *E. coli* S17-1λ*pir* (donor) and mobilised by conjugal mating into parental, ∆*cpxA* and ∆*cpxR* strains of *Yptb*. Genome integrated single copies of each Gfp-reporter fusion in recipient *Yptb*-YPIII strains was confirmed by colony PCR using respective genomic-integration primer pairs (Supplementary Table 2).

### Serial dilution-based biofilm dynamic assay

To assay for dynamic of biofilm formation in a plastic microtiter dish, a Crystal violet based serial dilution method was employed with modifications^98^. Briefly, optical density at 600 nm wavelength (OD_600_) of the overnight grown cultures was measured using a DU^®^ 730 Life Science UV/Vis spectrophotometer (Beckman Coulter). The number of cells were standardised to an OD_600_ of 0.1 using LB broth and then serially diluted 6-fold a total of nine times in sterile Eppendorf tubes. A volume of 150 μL from each serially diluted tube along with undiluted first tube was seeded in triplicate in the sterile 96-well round-bottom microtiter plate (Nunclon^TM^ ∆ Surface, Denmark). LB broth devoid of any bacteria was seeded in two separate columns as a negative control. The microtiter plates were incubated at 26 °C for 24 h with gentle agitation at 125 rpm. A duplicate plate was prepared and used for measuring the planktonic growth (OD_600_ nm). To determine if low bacterial concentration was the cause of an absence of biofilm formation, the initial bacterial-inoculum concentration was increased to an OD_600_ of 1.0. On the following day, seeded microtiter plates for biofilm biomass measurement were washed repeatedly with tap water and passed over an open Bunsen burner flame for 2–3 s to heat-fix the biofilm biomass. Biomass was stained with 200 µL of 0.5% (w/v) Crystal violet stain (Sigma Aldrich), and plates were incubated at room temperature for 15 min. The unbound stain was removed by repeated gentle rinsing with tap water. Crystal violet stained biofilm biomass was solubilised with 200 µL of 33% (v/v) Glacial acetic acid (Sigma Aldrich). Plates were incubated at 26 °C for 20 min with gentle agitation. Solubilised biofilm biomass (150 µL) was transferred into a sterile 96-well flat-bottom microtiter plate (Nunclon^TM^ ∆ Surface, Denmark) and absorbance measured at 570 nm using a TECAN spectrophotometer plate reader. In parallel, viability of bacteria from each seeded well was monitored before and after the formation of biofilm by spotting 3.5 µL culture from each on selective LA plates containing desired antibiotic(s) and further incubation at 26 °C for 24 h.

### Biofilm analysis on the surface of nematode, *Caenorhabditis elegans*

The N_2_ Bristol strain of nematode *Caenorhabditis elegans* was used throughout. The *C. elegans* were maintained on lawns of *E. coli* OP50 at room temperature in Petri dishes (55 mm diameter) containing NGM agar (Per liter: 17 g Difco bacto-agar, 25 g Difco bactopeptone, 0.3% NaCl, 0.5% Cholesterol, 1 M CaCl_2_, 1 M MgSO_4_ and 1 M Postssium phosphate buffer- pH 6.0)^99^. For the biofilm assay, an equal number of *Yptb* bacteria tagged with Gfp, expressed from pFPV25.1 (a gift from Raphael Valdivia via Addgene plasmid # 20668; http://n2t.net/addgene:20668; RRID:Addgene_20668) in 50 µL volumes (from overnight cultures equalised to the lowest OD_600_ measurement) were seeded on the NGM agar plates and incubated at 26 °C overnight. Thirty L4-stage (larval) of nematodes were transferred to the NGM plates containing the bacterial lawns and left at room temperature. Development of biofilm was examined by Gfp-expressing *Yptb* on the surface of the *C. elegans* every 24 h using fluorescence microscopy. Scoring of at least 30 live worms was carried out continuously for 5 days. This required that worms were transferred on sterile glass coverslips, rinsed gently with Phosphate buffered saline, and fixed in 50% sterile glycerol and viewed with Gfp-fluorescence at ×50 magnification, equivalent to a 10 µm scale bar.

### Gfp reporter assay

A bacterial inoculum was prepared from a single isolated colony and then grown in selective LB broth at 26 °C for 18 h with gentle agitation. On the following day, a sterile 96-well flat-bottom µCLEAR^®^ black polystyrene microtiter plate (Greiner Bio-one, Germany) was seeded (in triplicate) with 150 μL of an overnight inoculum standardised to an OD_600_ of 0.01. The lid of the plate was sealed with sterile Parafilm, and the plate was incubated at 26 °C for 24 h with gentle agitation at 125 rpm. LB broth devoid of any bacteria was seeded in two separate columns as a negative control. Extent of bacterial growth at a wavelength of 600 nm, and and Gfp-fluorescence (excitation ƛ 485 nm and emission ƛ 515 nm) were recorded after 24 h by a TECAN spectrophotometer with following kinetic settings established via the Infinite-200 Tecan i-control software (version 1.12.4.0): shaking (linear) every 5 min for 3 s, number of flashes-25, bandwidth- 9 nm for Absorbance A600 nm and fluorescence-excitation, and for fluorescence-emission- 20 nm, integration time- 20 µs, Gains- 70 and 90. Readings for both growth and Gfp-fluorescence were recorded from the top of the plate. The mean of gained Gfp-fluorescence was normalised with corresponding growth (OD_600_ nm) and calculated Gfp-fluorescence unit (FU/OD).

### Real-time qPCR

Total RNA from cultures standardised to the lowest OD_600_ was isolated using the Nucleo Spin RNA isolation kit (Macherey-Nagal, Germany) as per the manufacturer’s protocol. Isolated total RNA was treated with Turbo DNase (Thermo Scientific) and inactivated as recommended using DNase inactivation reagent. Turbo DNase treated total RNA template (200 ng) was used to synthesise cDNA using RevertAid H-minus reverse transcriptase (Thermo Scientific) as per the manufacturer’s protocol. A negative reaction (without reverse transcriptase) was also prepared to further check for DNA contamination. Both reverse transcriptase + /− reactions were incubated in PCR machine at 25 °C for 10 min, 42 °C for 60 min, 70 °C for 10 min and 4 °C for 10 min. Synthesised cDNA was quantified by a Nano-drop spectrophotometer (Thermo Scientific) and stored at −20 °C. Real-time qPCR was performed on iQ5 Thermocycler (Bio-Rad) using 50 ng of cDNA template combined with 1× qPCRBIO SyGreen mix with fluorescein and 400 nM of each gene-specific qRT-primer pair (Supplementary Table 2). PCR reactions (20 µL size) were performed as per the manufacturer’s protocol, considering melting temperature of respective primers pair and the in-built melting analysis for optimum annealing of each primer. Relative expression of each gene was calculated as 2^Λ^^−^^∆∆Ct^^100^. The expression of each gene was examined from three biological replicates having three technical replicates. To enhance the reliability of expression, each targeted gene was normalised with two house-keeping internal standards, *gyrB* and *rpoC*.

### Purification of wild-type and phosphorylation defective CpxR

An established recombinant *E*. *coli* BL21(DE3) pLysS strain expressing pKECO17 plasmid^64^ that contains *cpxR* gene from the parental *Yptb*-YPIII, cloned under the control of IPTG-inducible promoter of pET22b plasmid (Invitrogen) was used to express and purify C-terminally His_6_-tag fused CpxR_wt_. The phosphorylation defective CpxR_Pneg_ was designed by substituting Asp8, Asp9, Asp51, Met53 and Lys100 with Ala amino acid on *Yptb*-YPIII *cpxR* synthetic DNA (obtained from GenScript, USA) and subcloned into pET22b plasmid (Invitrogen). Subsequent expression, purification and storage of phosphorylation defective CpxR_Pneg_ was carried out by the Protein Expertise Platform (Umeå University, Sweden). Briefly, an overnight culture was prepared in 15 mL selective LB broth at 37 °C for 16 h. On the following day, 250 mL selective LB broth was sub-cultured (1/20) with overnight inoculum and grown for 2.5 h at 37 °C with aeration. The culture was induced with IPTG at a final concentration of 0.5 mM and the incubation continued for a further 4 h at 30 °C. Bacterial cells were harvested by centrifugation at 8000 × *g* for 15 min and mixed into 50 mL lysis buffer (20 mM Tris-HCl pH 7.5, 500 mM NaCl, 30 mM imidazole, 0.9% Triton X-100, 1% Glycerol, 8.75 mM β-mercaptoethanol and 5 mM EDTA-free PMSF proteases inhibitor). Cell lysate was prepared by sonication (pulse on/off- 10 sec, Amp- 50%) for 5 min ×4 and clarified by ultracentrifugation at 104,000 × *g* for 60 min at 4 °C to isolate soluble ‘active’ CpxR_wt_ and ‘inactive’ CpxR_Pneg_ from the supernatant fraction. The CpxR variants were purified manually using 1 mL HisTrap^TM^ HP column (GE Healthcare) as per the manufacturers protocol. Five protein fractions (1 mL each) were eluted sequentially with 150, 250 and 350 mM imidazole in the column binding buffer (20 mM Tris-HCl pH 7.5, 500 mM NaCl) and analysed by SDS-PAGE followed by Coomassie blue staining. Based on Coomassie blue staining, fraction 7 contained sufficiently pure CpxR_His6_, and was dialysed in a D-tube dialyser (Novagen, MWCO 6-8 kD) against dialysis buffer (10 mM Tris-HCl pH 7.5, 50 mM KCl and 1.0 mM β-mercaptoethanol) overnight at 4 °C with three changes of the dialysis buffer. Dialysed CpxR_His6_ was quantified by Nanodrop spectrophotometer (Thermo Scientific) and by classical Bradford assay. Glycerol (2.5%) and EDTA-free PMSF proteases inhibitor (1.0 mM) were added to the quantified CpxR variants and stored in aliquots at −20 °C.

### Electrophoretic mobility shift assay

DNA fragments encompassing the entire promoter regions of the target genes, and for control purposes the non-specific 16S rDNA (148 bp from YPK_R0086; *rRNA*), were PCR-amplified from the genomic DNA of parental *Yptb*-YPIII using EMSA primers, listed in Supplementary Table 2. The amplified DNA fragments were analysed by agarose gel electrophoresis and purified using the GenJET PCR purification kit (Thermo Scientific). For EMSA, either 50 µM or 100 µM of dialysed CpxR_wt_ or CpxR_Pneg_ variant was combined with 40 ng of non-specific 16S rDNA and 40 ng of target promoter fragment in EMSA binding buffer (50 mM Tris-HCl pH 8.0, 30 mM NaCl, 20 mM Acetyl-P and 1% β-mercaptoethanol). EMSA reactions (10 µL) were incubated for 30 min at 30 °C without agitation. Completed reactions were mixed with 2.5 µL of 5× EMSA loading dye (50 mM Tris-HCl pH 6.8, 0.01% Bromophenol blue, 25% glycerol) and 10 µL aliquots were loaded on homemade 1.0 mm thick 6% native Polyacrylamide gel [30% Acrylamide (37.5:1) with 2.6% cross-linker (1. 2 mL), 50% glycerol (300 µL), 1× TBE (300 µL), ddH_2_O (4.2 mL), 10% APS (60 µL) and TEMED (6.0 µL)]. The gel was electrophoresed in 1× TBE buffer at 100 volts at room temperature until the dye front had migrated to the end of the gel. Gels were subsequently stained with 1× GelRed (Cambridge Bioscience, UK), diluted in 1× TBE, at room temperature for 30 min with gentle agitation. The excessive stain was removed by with three successive 10 min washes with sterile ddH_2_O. The gel was imaged using GelDoc2000 (Bio-Rad) imager with an automatic exposure of UV-light.

### Congo Red-binding colony morphotype on salt-free Luria agar

The *Yptb*-YPIII strains were routinely grown in 2.5 mL LB broth lacking NaCl with shaking (150 rpm) at 26 °C-18 h. On the following day, an equal number of cells in 3.5 µL volumes (equalised to the lowest OD_600_ measurement) were spotted on LA (without NaCl) plates, supplemented with 0.01% (w/v) Congo Red (Sigma Aldrich) and appropriate antibiotic(s). Plates were incubated at 26 °C-24 h. Colony morphotype was imaged on an inverted microscope with an epi-light source.

### Phos-tag^TM^ of in vivo accumulated CpxR~P in the biofilm-planktonic cells

Equal numbers of cells were harvested from the desired *Yptb-*YPIII strains, grown in LB broth to late-stationary phase at 26 °C, in 96-well round-bottomed microtiter plate. This was achieved by combining planktonic culture from 8 wells of respective strain from the mature stage of biofilm (equivalent to 4^th^ dilution of the 6-fold dilution series). Harvested cells (by centrifugation at 16,000 × *g* for 20 min) were mixed in 100 μL of BugBuster^®^ Master Mix (Novagen^®^, Merck Millipore, Sweden) and cell lysis was performed by incubation at 26 °C for 30 min with gentle agitation. Cell lysis was halted by adding 100 μL 2x SDS-PAGE sample buffer lacking β-Mercapoethanol (100 mMTris-HCl; pH 6.8, 4% SDS, 20% Glycerol and 0.01% Bromophenol blue). Following heat-denaturation at 95 °C for 5 min, 5 μL total cell-lysate was electrophoresed on a freshly prepared 12% Phos-tag^TM^ Acrylamide AAL-107 gel (Wako Nard Institute, Japan) at 80 volts at room temperature, until dye front reaches at the bottom of gel. Preparation of Phos-tag^TM^ gel and subsequent processing for Western immunoblotting were as per the manufacturer’s suggestions. Following wet electrotransfer onto PVDF membrane (53 volts for 2 h at 4 °C), the two CpxR isoforms were bound with rabbit polyclonal anti-CpxR antibody (1: 2000 dilutions in TBST plus 5% skimmed milk) for overnight at 4 °C, followed by anti-rabbit-HRP antibody (1: 6000 in TBST plus 5% skimmed milk) for 1 h at room temprature, and then detected with Pierce^TM^ ECL Plus Western blotting system as per the manufacturer’s instructions using ImageQuant™ LAS 4000 imager (GE Healthcare).

### Quantification and statistical analysis

The level of active phosphorylated CpxR~P on Phos-tag^TM^ Western blot images was quantified using ImageJ^101^. The active level of CpxR~P was inferred as percentage as follow: Protein band intensity of CpxR~P divided by Protein band intensity of CpxR~P plus full-length CpxR at ~26.5 kDa, and multiplied by 100. Throughout, standard error of mean ± was calculated for at least three biological replicates. Significance from the parent or negative control was determined using One-way ANOVA with Tukey’s multiple comparisons test, with a single pooled variance. The difference in variance with a *p*-value of < 0.05 was considered significant. Analysis was performed using GraphPad Prism-7, for MacBook Pro (GraphPad Software, Inc. La Jolla, CA, USA).

### Reporting summary

Further information on research design is available in the Nature Research Reporting Summary linked to this article.

## Supplementary information

SUPPLEMENTAL MATERIAL

Updated Reporting Summary.

## Data Availability

The datasets generated and/or analysed during the current study are available from the corresponding authors on reasonable request. The NCBI database codes for retrieving the whole genome sequence of *Yersinia pseudotuberculosis* YPIII and *Yersinia pestis* KIM10 + are CP000950.1 and AE009952.1, respectively. The locus tag of the studied genes of *Y. pseudotuberculosis* YPIII are, YPK_4133 (*cpxA*), YPK_4132 (*cpxR*), YPK_4131 (*cpxP*), YPK_2241 (*hmsH*),YPK_3638 (*hmsT*), YPK_0094 (*hmsP*), YPK_3615 (*hmsC*), YPK_2238 (*hmsS*), YPK_1182 (*rpoE*), YPK_1090 (*nlpE*), YPK_1551 (*ackA*), YPK_1550 (*pta*), YPK_0004 (*gyrB*), YPK_0341 (*rpoC*) and YPK_R0086 (16 S rDNA). Software used for image quantification was ImageJ^101^. Software used for statistical analysis was contained within GraphPad Prism-7, for MacBook Pro (GraphPad Software, Inc. La Jolla, CA, USA).
