# Supplementary material for: Cpx-signalling facilitates Hms-dependent biofilm formation by Yersinia pseudotuberculosis
Source: NPJ Biofilms Microbiomes. 2022 Mar 29;8:13. doi: 10.1038/s41522-022-00281-4 (PMC8964730; doi:10.1038/s41522-022-00281-4)
Supplement: Supplementary file 1 — SUPPLEMENTAL MATERIAL [file 41522_2022_281_MOESM1_ESM.pdf]

SUPPLEMENTARY INFORMATION

Cpx-signalling facilitates Hms-dependent biofilm formation by *Yersinia pseudotuberculosis*

Dharmender K. Gahlot[1,2*], Sun N. Wai[1,2,3], David L. Erickson[4], Matthew S. Francis[1,2*]

[1]Department of Molecular Biology, Umeå University, Umeå, Sweden
[2]Umeå Centre for Microbial Research, Umeå University, Umeå, Sweden
[3]The Laboratory for Molecular Infection Medicine, Umeå University, Umeå, Sweden
[4]Department of Microbiology and Molecular Biology, Brigham Young University, Provo, UT, United States

*Correspondence to Dharmender K. Gahlot: dharmender.kumar@umu.se and Matthew S. Francis: matthew.francis@umu.se

The file contains the following information:

Tables:
Supplementary Table 1. Bacterial strains and plasmids used in this study.
Supplementary Table 2. Oligonucleotides used in this study.

Figures:
Supplementary Figure 1: Viability of bacteria cultured on an abiotic surface.
Supplementary Figure 2: *In vivo* accumulated CpxR~P in bacteria grown on an abiotic surface.
Supplementary Figure 3: Cpx-signalling mediates differential transcriptional regulation of *hms* loci.
Supplementary Figure 4: Genetic organisation of *rpoE* in the *rseAB* operon that codes for negative regulators of *rpoE*.
Supplementary Figure 5: Genetic conservation of CpxAR and the CpxP and NlpE auxiliary factors.
Supplementary Figure 6: *Y. pseudotuberculosis hmsHFRS* regulatory region is similar in *Y. pestis*.
Supplementary Figure 7: *Y. pseudotuberculosis hmsT* regulatory region is similar to *Y. pestis*.
Supplementary Figure 8: *Y. pseudotuberculosis hmsCDE* regulatory region is similar to *Y. pestis*.
Supplementary Figure 9: *Y. pseudotuberculosis hmsP* regulatory region is similar to *Y. pestis*.

Unprocessed (raw) image of immunoblots and EMSA-gels corresponding to the relevant figures are included at the end of this document.

Supplementary Table 1. Bacterial strains and plasmids used in this study.

**Strain or plasmid**	**Relevant genotype or phenotype**[1]	**Source or reference**
E. coli **strains**		
DH5á	F^{-}, *recA*1, *endA*1, *hsdR*17, *supE*44, *thi*-1, *gyrA*96, *relA*1	Vicky Shingler
SY327*λpir*	F^{-}, *araD*, Δ(*lac pro*), *argE*(Am), *recA56*, Rif^{R}, *gyrA λpir*	(1)
S17-1*λpir*	*recA*, *thi*, *pro*, *hsdR*$^{-}$*M*$^{+}$, Sm^{R}, <RP4:2-Tc:Mu:Ku:Tn7>Tp^{R}	(2)
BL21(DE3) plysS	F^{-}, *dcm*, *lon*, *ompT*, *hsdS*(r_B^-, m_B^-), *gal*, λ(DE3), [pLysS, Cml^{R}]	Promega
Y. pseudotuberculosis strains		
WT (YPIII/pIB102)	*yadA*::Tn5, PhoP^{-}; Km^{R}	(3)
Δ*cpxA* (YPIII07/pIB102)	pIB102, *cpxR* in frame deletion of codons 41 to 449; Km^{R}	(4)
Δ*cpxR* (YPIII08/pIB102)	pIB102, *cpxR* in frame deletion of codons 11 to 139; Km^{R}	(4)
Δ*cpxP* (YPIII41/pIB102)	pIB102, *cpxP* in frame deletion of codons 15 to 151; Km^{R}	This study
Δ*hmsS* (YPIII_2238/pIB102)	pIB102, *hmsS* in frame deletion of codons 11 to 135; Km^{R}	This study
Δ*nlpE* (YPIII34/pIB102)	pIB102, *nlpE* in frame deletion of codons 12 to 213; Km^{R}	This study
$CpxR_{Pneg}$ (YPIII_4132pneg/pIB102)	pIB102, $cpxR_{D8A, D9A, D51A, M53A, K100A}$; Km^{R}	This study
Δ*ackA*, *pta* (YPIII69/pIB102)	pIB102, *ackA*, *pta* in frame double deletion from codon 17 of *ackA* through to codon 680 of *pta*; Km^{R}	(5)
Δ*ackA*, *pta*, *cpxA* (YPIII49/pIB102)	*ackA*, *pta* in frame double deletion introduced into YPIII07/pIB102; Km^{R}	(5)
**Plasmids**		
pJET1.2/Blunt	CloneJET PCR cloning and sequencing vector, Amp^{R}	Thermo Scientific
pDM4	Suicide plasmid carrying *sacBR;* Cml^{R}	Debra Milton
pMF683	*Xho*I/*Xba*I PCR fragment of Δ*cpxP* in pDM4; Cm^{R}	This study
pDKG01	*Xho*I/*Xba*I PCR fragment of Δ*hmsS* in pDM4; Cm^{R}	This study
pKEC007	*Xho*I/*Xba*I PCR fragment of Δ*nlpE* in pDM4; Cm^{R}	This study
pDM-DK1011	*Xho*I/*Xba*I PCR fragment of $cpxR_{D8A, D9A, D51A, M53A, K100A}$ in pDM4; Cm^{R}	This study
pNQ705-1	GFPmut3* based promoter reporter; Cml^{R}	Debra Milton
pDK2241::Gfp	708 bp *Sac*I/*Sph*I PCR fragment of P_{hmsH} in pNQ705-1; Cml^{R}	This study
pDK3638::Gfp	315 bp *Sac*I/*Sph*I PCR fragment of P_{hmsT} in pNQ705-1; Cml^{R}	This study

pDK0094::Gfp	378 bp *Sac*I/*Sph*I PCR fragment of P_{hmsP} in pNQ705-1; Cml^R	This study
pWKS30	Low copy number cloning plasmid; Amp^R	(6)
pJF067 (pCpxA_{wt})	1549 bp synthetic DNA fragment of *cpxA* (full length) with its native promoter cloned into *Xba*I-*Xho*I digested pWKS30; Amp^R	(7)
pMMB208	Expression plasmid; Cml^R	(8)
pKEC021	747 bp *Xba*I/*Kpn*I PCR fragment of *cpxR* in pMMB208; Cml^R	(9)
pDK1007 (pCpxR_{Pneg})	747 bp *Xba*I/*Kpn*I PCR fragment of *cpxR*$_{D8A, D9A, D51A, M53A, K100A}$ in pMMB208; Cml^R	This study
pDK1011 (pCpxR_{Pneg})	1032 bp *XhoI*/*Xba*I PCR fragment of *cpxR*$_{D8A, D9A, D51A, M53A, K100A}$ and flanking DNA up-and downstream cloned in pUC57; Ap^R	This study
pET22b(+)	Expression vector; Amp^R	Novagen
pKEC017	~700 bp *Nde*I/*Xho*I PCR fragment of *cpxR* in pET22b(+) that creates a $His_{(6)}$ C-terminal fusion; Amp^R	(9)
pFVP25.1	GFP expression plasmid, driven by constitutive promoter from *Salmonella*; Amp^R	(10)

[1]Sm^R: Streptomycin-resistant, Tp^R: Trimethoprim-resistant, Cml^R: Chloramphenicol-resistant, Km^R: Kanamycin-resistant, Amp^R: Ampicillin-resistant, $Rif^{R:}$ Rifamycin-resistant

Supplementary References

1. Miller VL, Mekalanos JJ. A novel suicide vector and its use in construction of insertion mutations: osmoregulation of outer membrane proteins and virulence determinants in *Vibrio cholerae* requires toxR. J Bacteriol. 1988;170(6):2575-83.

2. Simon R, Priefer U, Pühler A. A broad host range mobilisation system for *in vivo* genetic engineering: transposon mutagenesis in Gram negative bacteria. Biotechnology. 1983;1:787-96.

3. Wolf-Watz H, Portnoy DA, Bolin I, Falkow S. Transfer of the virulence plasmid of *Yersinia pestis* to *Yersinia pseudotuberculosis*. Infect Immun. 1985;48(1):241-3.

4. Carlsson KE, Liu J, Edqvist PJ, Francis MS. Extracytoplasmic-stress-responsive pathways modulate type III secretion in *Yersinia pseudotuberculosis*. Infect Immun. 2007;75(8):3913-24.

5. Liu J, Obi IR, Thanikkal EJ, Kieselbach T, Francis MS. Phosphorylated CpxR restricts production of the RovA global regulator in *Yersinia pseudotuberculosis*. PLoS One. 2011;6(8):e23314.

6. Wang RF, Kushner SR. Construction of versatile low-copy-number vectors for cloning, sequencing and gene expression in *Escherichia coli*. Gene. 1991;100:195-9.

7. Thanikkal EJ, Gahlot DK, Liu J, Fredriksson Sundbom M, Gurung JM, Ruuth K, et al. The *Yersinia pseudotuberculosis* Cpx envelope stress system contributes to transcriptional activation of *rovM*. Virulence. 2019;10(1):37-57.

8. Morales VM, Backman A, Bagdasarian M. A series of wide-host-range low-copy-number vectors that allow direct screening for recombinants. Gene. 1991;97(1):39-47.

9. Carlsson KE, Liu J, Edqvist PJ, Francis MS. Influence of the Cpx extracytoplasmic-stress-responsive pathway on *Yersinia* sp.-eukaryotic cell contact. Infect Immun. 2007;75(9):4386-99.

10. Valdivia RH, Falkow S. Bacterial genetics by flow cytometry: rapid isolation of *Salmonella typhimurium* acid-inducible promoters by differential fluorescence induction. Mol Microbiol. 1996;22(2):367-78.

11. De Wulf P, McGuire AM, Liu X, Lin EC. Genome-wide profiling of promoter recognition by the two-component response regulator CpxR-P in *Escherichia coli*. J Biol Chem. 2002;277(29):26652-61.

12. Yamamoto K, Ishihama A. Characterization of copper-inducible promoters regulated by CpxA/CpxR in *Escherichia coli*. Biosci Biotechnol Biochem. 2006;70(7):1688-95.

13. Fang N, Yang H, Fang H, Liu L, Zhang Y, Wang L, et al. RcsAB is a major repressor of *Yersinia* biofilm development through directly acting on *hmsCDE*, *hmsT*, and *hmsHFRS*. Sci Rep. 2015;5:9566.

Supplementary Table 2. Oligonucleotides used in this study[1]

Target gene	Oligonucleotides pair (5′-3′)	
**pDM4-mediated gene deletion mutagenesis**		
Δ*cpxP*	p*cpxP*A	**CTCGAG**GTCATCATCAACTAATAGGA ***Xho*I**
(Δ15-151 aa)	p*cpxP*B	AACGAACATTGACGCCATAAC
	p*cpxP*C	ATGGCGTCAATGTTCGTTAAACCAGCAGCACAACCTTC
	p*cpxP*D	**TCTAGA**AGGGTGGATTAGCTGCC ***Xba*I**
Δ*hmsS*	YPK_2238A	GACC**CTCGAG**ATGGTATTGGCACTGACATGTC ***Xho*I**
(Δ11-135 aa)	YPK_2238B	CTGTTCCGTAAAAATCAGGGGCGTACTC
	YPK_2238C	CCCCTGATTTTTACGGAACAGGTTGCTCCAGTGATCCATTTACGCC
	YPK_2238D	CCCT**TCTAGA**CACTGACGGAGTGAATACCTC ***Xba*I**
Δ*nlpE*	p*nlpE*A	**CTCGAG**GCCAGGCAATGGTGGTAG ***Xho*I**
(Δ12-213 aa)	p*nlpE*B	TGCCAAAAGTAGCGTTATCGT
	p*nlpE*C	ACGATAACGCTACTTTTGGCAGGGGATATTCAGTTTAATGCC
	p*nlpE*D	**TCTAGA**ACAAATGATTAAGACCAGCG ***Xba*I**
**GFP-translational reporter fusion in pNQ-705-1 plasmid**		
$P_{hmsH::Gfp}$	YPK_2241F	CTC**GAGCTC**CATTGTATCGTAGC ***Sac*I**
	YPK_2241R*	CAT**GCATGC**TCAGTAGTGTTGTAAATGC ***Sph*I**
$P_{hmsT::Gfp}$	YPK_3638F	CTC**GAGCTC**CATATATCACTCTTTGG ***Sac*I**
	YPK_3638R*	CAT**GCATGC**TAAGCTGATCGTAGGAG ***Sph*I**
$P_{hmsP::Gfp}$	YPK_0094F	CTC**GAGCTC**GTCAACGTTAAGATTTCAG ***Sac*I**
	YPK_0094R*	CAT**GCATGC**TTTGTTTAATCGTTAATGAGCG ***Sph*I**
**To confirm genomic integration of GFP-translational reporter fusions**		
$P_{hmsH::Gfp}$	YPK_2241IF	CTTAAGCCTTGAAACGCCTTATTAC
	GfpR2	CATTCTTTTGTTTGTCTGCCATG
	YPK_2241IR	CCTGTAAATAGTCCAAGACTGG
	CatR2	AACACTATCCCATATCACCAGC
$P_{hmsT::Gfp}$	YPK_3638IF	GAACGGTAAGCTGCAAATGTCG
	GfpR2	CATTCTTTTGTTTGTCTGCCATG
	YPK_3638IR	GCGACGATGATAGCATACTG
	CatR2	AACACTATCCCATATCACCAGC
$P_{hmsP::Gfp}$	YPK_0094IF	CAGAGCCATTTAATCAGCAAACAC
	GfpR2	CATTCTTTTGTTTGTCTGCCATG
	YPK_0094IR	CTTTCCAACTGGTTGGCATAATC
	CatR2	AACACTATCCCATATCACCAGC
**Sequencing primers**		
	pJET1.2F	CGACTCACTATAGGGAGAGCGGC
	pJET1.2R	AAGAACATCGATTTTCCATGGCAG
	R6KR	ACAGGGTTGAACTGGCTGGATCTCC
	DK1011-12A	TGACGCTGCTGTTCAGTGAGGTTG
	DK1011-12B	CATTAAACGGTGCTGCCAGATAATCATC
	DK1011-12C	GATGATTATCTGGCAGCACCGTTTAATG
	DK1011-12D	TCAGCACTAGCATGAGCACTAACG
**qRT-PCR**		
hmsH	2241-qRTf	GCTGAGCATTATCGCCAAC
	2241-qRTr2	GATAATCCGCTAACGCCTTATC
	Amplicon size 121 bp	

hmsT	3638-qRTf ATCATCGTCGCCCAAGACAC
	3638-qRTr2 TAGTTATTTCTGCCCGCGTCG
	Amplicon size 120 bp
hmsP	0094-qRTf GCCCTTAGCGCATTTAGTC
	0094-qRTr2 TACACCAAGCGATAGATACC
	Amplicon size 131 bp
hmsC	3615-qRTf CATTATTGGTGGCTGTACCAACC
	3615-qRTr CCAGCGTGAATAACTGATAATACC
	Amplicon size 122 bp
rpoE	qRT-1182f ACTGGTCATTCGCTACCAGC
	qRT-1182r CCACGAAATGACTCCAGTGC
	Amplicon size 126 bp
gyrB	0004-qRTf GGACAACGCTATTGACGAAGC
	0004-qRTr CCTTCCTCATCGTGCATACC
	Amplicon size 129 bp
rpoC	0341-qRTf CTTCTCCAACTGCGCACATC
	0341-qRTr GACGTTCCAGGTTGGTCATAC
	Amplicon size 147 bp
**EMSA**	
P_{hmsH}	EMSA-2241F AACTGGATCGTGACTTACGTCG
	EMSA-2241R GCAGTAGTGTTGTAAATGCG
	Promoter size 728 bp
P_{hmsT}	EMSA-3638F ATATCACTCTTTGGCACTC
	EMSA-3638R AATATCGTGCTGTCAGTAG
	Promoter size 248 bp
P_{hmsP}	EMSA-0094F TGGCACCGTCAACGTTAAG
	EMSA-0094R ACTCGTTCTCCGCCAATTCC
	Promoter size 333 bp
P_{hmsC}	EMSA-3615F TAACATCAGTCAGAAGAG
	EMSA-3615R TCATTTTTGCGCTTCCTG
	Promoter size 381 bp
P_{rpoE}	EMSA-1182F CGTATGATGTGTGCAGAAG
	EMSA-1182R CGTTAACTGCTCGCTCATCC
	PCR product size 239 bp
16S rDNA	16SrDNAF4 TTTGTTGCCAGCACGTAATGGT
	16SrDNAR4 GCGAGTTCGCTTCACTTTGTATCT
	Fragment size 148 bp

[1]Primers were synthesised by Sigma-Aldrich Sweden AB, Stockholm, Sweden.

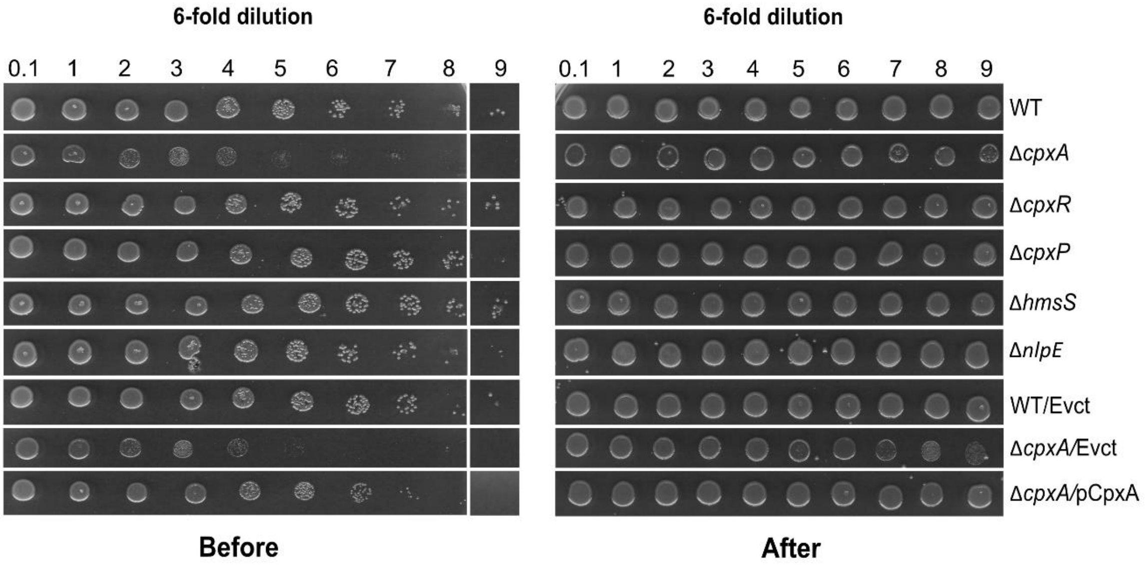

Supplementary Figure 1: Viability of bacteria cultured on an abiotic surface. Selective LB-agar was used to measure the viability of 3.5 μL bacterial inoculums in a serial dilution-based biofilm assay. Bacteria sampled at time point 0 h were taken immediately after inoculation into the plastic microtiter tray wells and are designated as "Before". Bacteria sampled at time point 24 h were taken immediately before the process of staining for biofilms with Crystal Violet and are designated as "After". Viability of each strain was monitored from three biological replicates, and representative data from one replicate is shown.

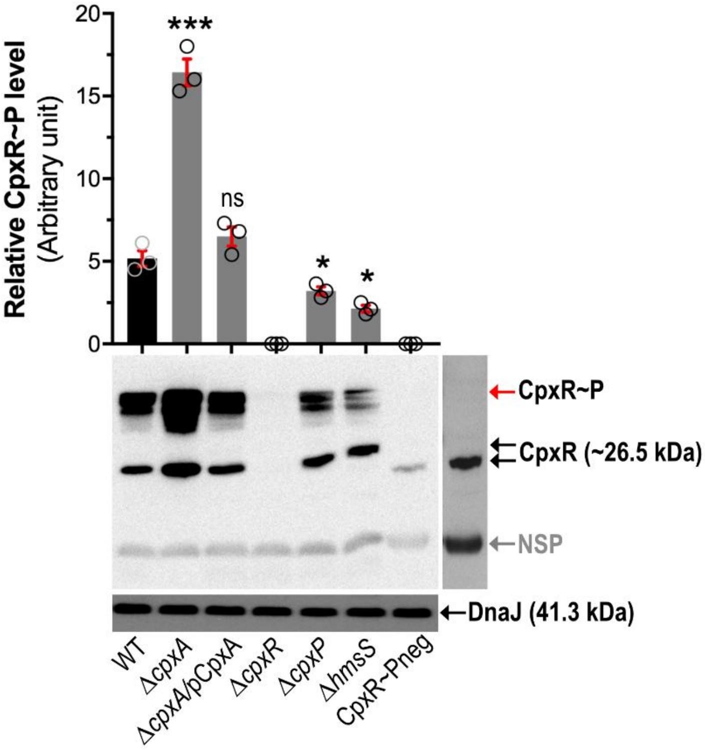

Supplementary Figure 2: ***In vivo* accumulated CpxR~P in bacteria grown on an abiotic surface.** The Phos-tag™ acrylamide system was used to measure accumulated CpxR~P in bacteria grown in LB broth to late stationary phase at 26 °C in 96-well round-bottomed microtiter plate. Samples were recovered from the planktonic portion of the culture from the mature stage of biofilm (equivalent to 4th dilution of the 6-fold dilution series). Lysed bacteria were electrophoresed on a freshly prepared 12% Phos-tag™ Acrylamide AAL-107 gel, immunoblotted, and detected with anti-CpxR antiserum. Assay specificity was validated using the CpxR-Pneg mutant variant that separated as only the non-phosphorylated isomer. For this variant, a phosphorylated form was not detectable, even after prolonged exposure of the immunoblot (right panel). The cytoplasmic molecular chaperone DnaJ served as a loading control. Strains: parent (WT), YPIII/pIB102; Δ*cpxA* null-mutant, YPIII07/pIB102; Δ*cpxA* null- mutant/pCpxA, YPIII07/pIB102, pJF067; Δ*cpxR* null-mutant, YPIII08/pIB102; Δ*cpxP* null-mutant, YPIII41/pIB102; Δ*hms* null mutant, YPIII_2238/pIB102; CpxR-Pneg mutant, YPIII_4132pneg/pIB102. The red arrow reflects the active phosphorylated CpxR isoform accumulated in the *Yersinia* cytoplasm, while the black arrow indicates the accumulated inactive non-phosphorylated CpxR isoform. An unknown degradation product (NSP; non-specific protein) is indicated by a grey arrow. A statistical significance between accumulated CpxR~P levels from the various strains with respect to the parent (WT) was determined using One-way ANOVA with Tukey's multiple comparisons test, with a single pooled variance. The difference in variance with a p-value of <0.05 was considered significant. The p-values are indicated by <0.001 (***) and >0.05 (ns; non-significant). Data is derived from three biological replicates. The representative unprocessed (raw) image of each blot can be seen at the end of this Supplementary file.

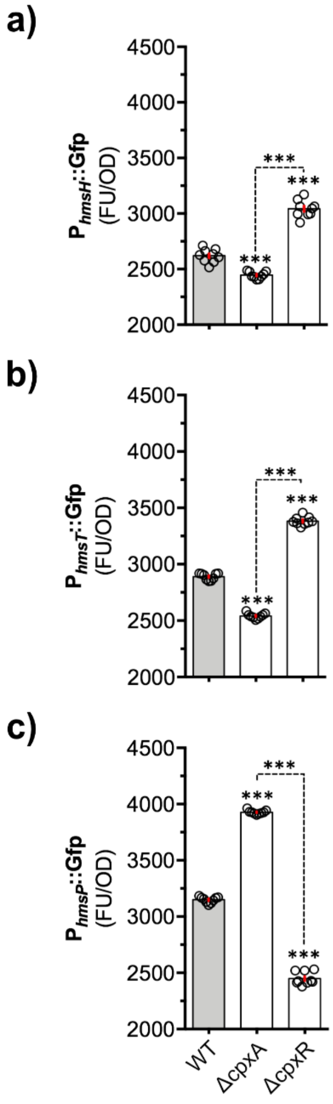

Supplementary Figure 3: Cpx-signalling mediates differential transcriptional regulation of *hms* loci. The translational fusions (**a**) *hmsH::Gfp,* (**b**) *hmsT::Gfp,* and (**c**) *hmsP::Gfp* were integrated *in cis* in the genome of parental (WT) (YPIII/pIB102), *ΔcpxA* null-mutant (YPIII07/pIB102), and *ΔcpxR* null-mutant (YPIII07/pIB102). Emitted fluorescence (excitation λ_{485} and emission λ_{515}) was recorded from cultures grown in a 96-well flat bottom µCLEAR® black polystyrene microtiter plate and standardise to an equal OD_{600} value after 24 h incubation with shaking at 150 rpm at 26 °C. The extent of Gfp-fluorescence (FU/OD) was calculated upon normalisation with culture density (OD_{600}). The FU/OD of each reporter strain in *ΔcpxA* and *ΔcpxR* was expressed as a value relative to WT**.** Error bars on the graphs represent standard error of mean (s. e. m) from three biological replicates routinely containing three technical replicates. Statistical significance with respect to the parent (WT) was determined using One-way ANOVA with Tukey's multiple comparisons test, with a single pooled variance. The difference in variance with a p-value of <0.05 was considered significant. The p-values are indicated by <0.001 (***).

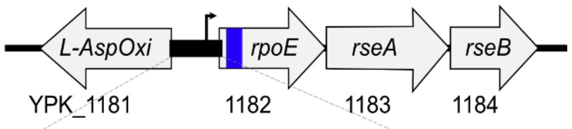

CGTATGATGTGTGCAGAAGAAGAGTTGGTTTCATGTTAGCCTATCTGCT

CAAGAAAAAGCTATGGGTATAAGATGTCTGAATAATATTTGGACAAAAA

-35 -10 +1
ATCGGGCA**ACA**TGGAAC**TTTAC**AATT**TTTA**T**AA**ACTCCA**A**GCGTGTGCT

TGCTCAGGAAACAATGATATGGCTGGCAGTACTGTTTTTAGCATGGGGA

RBS RpoE
TAGATTTCGGG**AGAGT**TCACCTCGG**ATG**AGCGAGCAGTTAACG

Supplementary Figure 4: Genetic organisation of *rpoE* in the *rseAB* operon that codes for negative regulators of *rpoE*. The locus-tag (YPK_XXXX) of each gene is mentioned underneath. Upstream gene, YPK_1181 codes for L-aspartate oxidase. PCR-amplified gene-specific (for qRT-PCR) and 5′ UTR (for EMSA) fragments are represented by respective vertical blue and horizontal black rectangle box. The P_{rpoE} EMSA fragment (zoomed out DNA sequence) from the upstream promoter region of *rpoE* of *Yptb*-YPIII shows potential CpxR~P binding motifs that would present on the antisense strand (yellow highlight), and the reverse complement of which is very similar to the established CpxR~P DNA-binding consensus sequence, 5′-GTAAA(N)$_{4\text{-}8}$ GTAAA-3′ (11, 12). Potential Sigma-70 based promoter elements, –35 and –10, are bold and underlined. The +1A identifies a potential transcriptional start site. The potential ribosomes binding site (RBS) upstream of ATG start codon is bold and underlined.

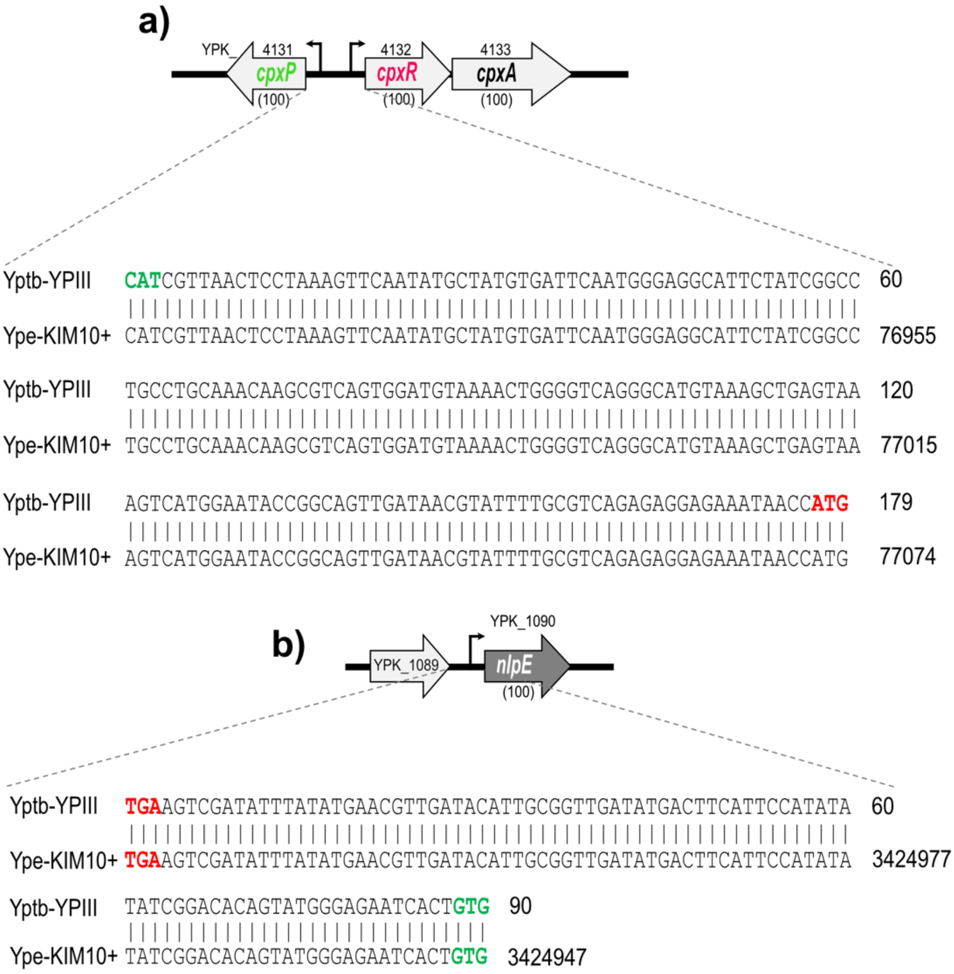

Supplementary Figure 5: Genetic conservation of CpxAR and the CpxP and NlpE auxiliary factors. Operon structure of *Y. pseudotuberculosis* Cpx-signalling system (**a**) and the auxiliary signalling molecule, NlpE (**b**). Part (**a**) shows the intergenic region of *cpxP* and *cpxR* (173 bp; zoomed out DNA sequence) and full-length protein coding sequence of *cpxP* (YPK_4131), *cpxR* (YPK_4132) and *cpxA* (YPK_4133) of Cpx-signalling from Y. *pseudotuberculosis* (*Yptb*-YPIII) was aligned with *Yersinia pestis* (*Ype*-KIM10+) whole genome (sequence ID: AE009952.1) using NCBI-BLAST. Similarly, part (**b**) shows the intergenic region upstream of *nlpE* (90 bp; zoomed out DNA sequence) and full-length protein coding sequence of *nlpE* (YPK_1090) from *Yptb*-YPIII and aligned with *Ype*-KIM10+. Entire sequence of Cpx-signalling from both *Yersinia* species reveals 100% identity at both genomic (pair-wise alignment) and protein (in the parenthesis) level. The start codons ATG (*cpxP* – shown as reverse complement CAT), ATG (*cpxR*) and GTG (*nlpE*) are coloured for illustration. Directionality of the promoter driving transcription of *cpxP*, *cpxRA* and *nlpE* is indicated by an arrow.

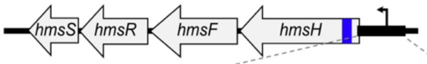


```
Yptb-YPIII  AACTGGATCGTGACTTACGTCGGTATTTCTAATTTATTCATTGTATCGTAGCCCGGATAA  60
            ||||||||||||||||||||||||||||||||||||||||||||||||||||||||||||
Ype-KIM10+  AACTGGATCGTGACTTACGTCGGTATTTCTAATTTATTCATTGTATCGTAGCCCGGATAA  2604033

Yptb-YPIII  AACAGATTCATCTAATTTTCGCTGGATCTGTTTTCATCGTGTCGCTCTGTCAGATGCTTA  120
            ||||||||||||||||||||||||||||||||||||||||||||||||||||||||||||
Ype-KIM10+  AACAGATTCATCTAATTTTCGCTGGATCTGTTTTCATCGTGTCGCTCTGTCAGATGCTTA  2603973

Yptb-YPIII  GATTTTAAAGTGGTAATAAATAGCGAGTTAAATTACGAAAGTTGTTTAATGACTTATTTC  180
            ||||||||||||||||||||||||||||||||||||||||||||||||||||||||||||
Ype-KIM10+  GATTTTAAAGTGGTAATAAATAGCGAGTTAAATTACGAAAGTTGTTTAATGACTTATTTC  2603913

Yptb-YPIII  GCTAGATTCTTTCTGTGATAATTAAAATTGTAATGATTCGATATATGATTATAACGTCAT  240
            ||||||||||||||||||||||||||||||||||||||||||||||||||||||||||||
Ype-KIM10+  GCTAGATTCTTTCTGTGATAATTAAAATTGTAATGATTCGATATATGATTATAACGTCAT  2603853

Yptb-YPIII  TGATAATCTACTTTGCTGAAGACTTGTCACGTTTGATTCACGATAATCATAGAAATGCAT  300
            ||||||||||||||||||||||||||||||||||||||||||||||||||||||||||||
Ype-KIM10+  TGATAATCTACTTTGCTGAAGACTTGTCACGTTTGATTCACGATAATCATAGAAATGCAT  2603793

Yptb-YPIII  AAAAAAAATTAATAATATTCAGTGCTAAAATCGATAAATGTCCCTTGAAATCCGTTCAGT  360
            ||||||||||||||||||||||||||||||||||||||||||||||||||||||||||||
Ype-KIM10+  AAAAAAAATTAATAATATTCAGTGCTAAAATCGATAAATGTCCCTTGAAATCCGTTCAGT  2603733

Yptb-YPIII  GAACAAATTACGCTGCTCTTCCAGCAGAACACTGTATCGCAGCATTCACTTAGTAAAAAT  420
            ||||||||||||||||||||||||||||||||||||||||||||||||||||||||||||
Ype-KIM10+  GAACAAATTACGCTGCTCTTCCAGCAGAACACTGTATCGCAGCATTCACTTAGTAAAAAT  2603673
                                -35                       -10       +1
Yptb-YPIII  ATTTACTCTTATTTTTTTGCATATTTAAGGGATATTAATCTAGGATTATTCTTAATTGCA  480
            ||||||||||||||||||||||||||||||||||||||||||||||||||||||||||||
Ype-KIM10+  ATTTACTCTTATTTTTTTGCATATTTAAGGGATATTAATCTAGGATTATTCTTAATTGCA  2603613

Yptb-YPIII  TTTTAATTAACGTCTTTGTTACATTTTCACCTCCATTAATCGCTGCAATAACAATATCCT  540
            ||||||||||||||||||||||||||||||||||||||||||||||||||||||||||||
Ype-KIM10+  TTTTAATTAACGTCTTTGTTACATTTTCACCTCCATTAATCGCTGCAATAACAATATCCT  2603553

Yptb-YPIII  ATAATGACTCTGCAACAATAATCCCTTTCAGCTAACCTATATGGCATGGTGTAATACATT  600
            ||||||||| |||||||||||||||||||||||||||||||||||||||| |||||||||
Ype-KIM10+  ATAATGACTTTGCAACAATAATCCCTTTCAGCTAACCTATATGGCATGGTATAATACATT  2603493

Yptb-YPIII  CTAAGAATTCCATTTCATCATCATTCCATAATGAAAAATGTACGTTCAGGAAGTCGCTTT  660
            ||||||||||||||||||||||||||||||||||||||||||||||||||||||||||||
Ype-KIM10+  CTAAGAATTCCATTTCATCATCATTCCATAATGAAAAATGTACGTTCAGGAAGTCGCTTT  2603433
                                         RBS        HmsH
Yptb-YPIII  CCTTGATCATCAAACAAGTTGCTGGCTTAAGGGTTATATAATGTATAACGCATTTACAAC  720
            ||||||||||||||||||||||||||||||||||||||||||||||||||||||||||||
Ype-KIM10+  CCTTGATCATCAAACAAGTTGCTGGCTTAAGGGTTATATAATGTATAACGCATTTACAAC  2603373

Yptb-YPIII  ACTACTGC  728
            ||||||||
Ype-KIM10+  ACTACTGC  2603363
```

Supplementary Figure 6: *Y. pseudotuberculosis hmsHFRS* regulatory region is similar in *Y. pestis*. Shown is the operon structure of *Y. pseudotuberculosis hmsHFRS* genes cluster. The $P_{hmsHFRS}$ EMSA fragment (thick horizonal black line; zoomed out DNA sequence) from the upstream promoter region of *hmsHFRS* operon of Y. *pseudotuberculosis* (*Yptb*-YPIII) was aligned with *Yersinia pestis* (*Ype*-KIM10+) whole genome (sequence ID: AE009952.1) using NCBI-BLAST. $P_{hmsHFRS}$ EMSA fragment reveals 99% genomic identity (pair-wise alignment) between the two sequences. Non-identical nucleotides are highlighted in grey. ATG identifies the start codon of *hmsH*. The promoter transcribing *hmsHFRS* operon is indicated by a directional arrow. A potential CpxR~P binding motif as per established consensus (11, 12) is partly on the sense strand and highlighted in green (5′-GTAAA-3′) and partly on the antisense strand and highlighted in yellow (reverse complement would be 5′-GTAAA-3′). Promoter elements, -10 and -35, +1 T transcriptional start site and ribosomes binding site (RBS) were recognised as per the publication of Fang and colleagues (13).

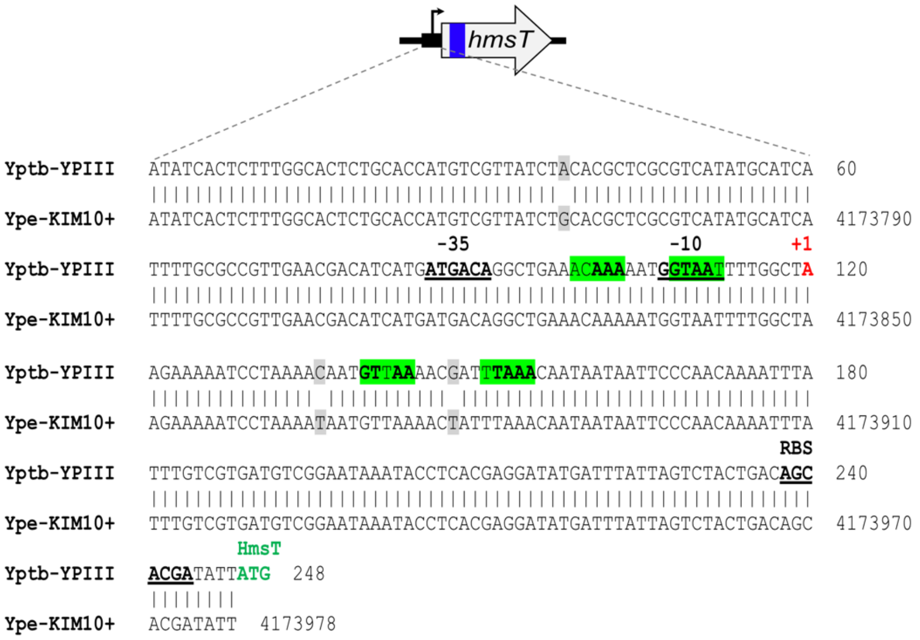

Supplementary Figure 7: *Y. pseudotuberculosis hmsT* regulatory region is similar to *Y. pestis*. Shown is the genetic organisation of *Y. pseudotuberculosis hmsT* gene. The P_{hmsT} EMSA fragment (thick horizonal black line; zoomed out DNA sequence) from the upstream promoter region of *hmsT* of Y. *pseudotuberculosis* (*Yptb*-YPIII) was aligned with *Yersinia pestis* (*Ype*-KIM10+) whole genome (sequence ID: AE009952.1) using NCBI-BLAST. P_{hmsT} EMSA fragment reveals 99% genomic identity (pair-wise alignment) between the two sequences. Non-identical nucleotides are highlighted in grey. The promoter transcribing *hmsT* is indicated by directional arrow. Potential CpxR~P binding motifs are highlighted in green based on their similarity to the established 5′-GTAAA(N)$_{4-8}$ GTAAA-3′ (11, 12). Promoter elements, -10 and -35, +1 A transcriptional start site and ribosomes binding site (RBS) were recognised as per the publication of Fang and colleagues (13).

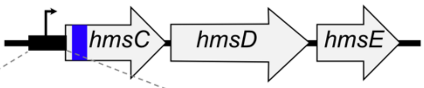


```
Yptb-YPIII    TAACATCAGTCAGAAGAGAAAAAGCCCCGGAGCAGTCCAGCCCAGCTCTGGGGCTTTTGA  60
              |||||||||||||||||||||||||||||||||||||||||||||||| |||||||||||
Ype-KIM10+    TAACATCAGTCAGAAGAGAAAAAGCCCCGGAGCAGTCCAGCCCAGCTCCGGGGCTTTTGA  4145957

Yptb-YPIII    GGTAAATAACCCCCGTGTATAGGCCAGTGAATGAAGGGTCTACTTCCCCTACTCGTGCTT  120
              ||||||||||||||||||||||||||||||||||||||||||||||||||||||||||||
Ype-KIM10+    GGTAAATAACCCCCGTGTATAGGCCAGTGAATGAAGGGTCTACTTCCCCTACTCGTGCTT  4146017

Yptb-YPIII    CAGTGGCTCATGTCATGACGCCCCCAACGGCACGTTTCACCTTTATCTGAGGTGATCAAC  180
              ||||||||||||||||||||||||||||||||||||||||||| ||||||||||||||||
Ype-KIM10+    CAGTGGCTCATGTCATGACGCCCCCAACGGCACGTTTCACCTTCATCTGAGGTGATCAAC  4146077

Yptb-YPIII    TACCTGACAAAAACCCGGAAAACAGTCGGGTTTTTTATATTTCTATTTCAATAACATAAA  240
              ||||||||||||||||||||||||||||||||||||||||||||||||||||||||||||
Ype-KIM10+    TACCTGACAAAAACCCGGAAAACAGTCGGGTTTTTTATATTTCTATTTCAATAACATAAA  4146137
                                                   -35                   -10
Yptb-YPIII    TAAGATTTAAGATAAATCTCAAATTTACGATGATATGACTATTATTACATCATATAAATC  300
              ||||||||||||||||||||||||||||||||||||||||||||||||||||||||||||
Ype-KIM10+    TAAGATTTAAGATAAATCTCAAATTTACGATGATATGACTATTATTACATCATATAAATC  4146197
                       +1
Yptb-YPIII    TTATAATAACGTCGTACCTAACGCCGATTCAACCACCACATTTAGCATCCCTTTTTAGCC  360
              ||||||||||||||||||||||||||||||||||||||||||||||||||||||||||||
Ype-KIM10+    TTATAATAACGTCGTACCTAACGCCGATTCAACCACCACATTTAGCATCCCTTTTTAGCC  4146257
                   RBS         HmsC
Yptb-YPIII    CAACAGGAAGCGCAAAAATGA  381
              |||||||||||| ||||||||
Ype-KIM10+    CAACAGGAAGCGTAAAAATGA  4146278
```

Supplementary Figure 8: *Y. pseudotuberculosis hmsCDE* regulatory region is similar to *Y. pestis*. Shown is the operon structure of the *Y. pseudotuberculosis hmsCDE* genes cluster. The P_{hmsCDE} EMSA fragment (thick horizonal black line; zoomed out DNA sequence) from the upstream promoter region of *hmsCDE* of Y. *pseudotuberculosis* (*Yptb*-YPIII) was aligned with *Yersinia pestis* (*Ype*-KIM10+) whole genome (sequence ID: AE009952.1) using NCBI-BLAST. P_{hmsCDE} EMSA fragment reveals 99% genomic identity (pair-wise alignment) between the two sequences. Non-identical nucleotides are highlighted in grey. The promoter transcribing *hmsCDE* is indicated by a directional arrow. ATG identifies the start codon of *hmsC.* A potential CpxR~P binding motif as per established consensus (11, 12) is partly on the sense strand and highlighted in green (5′-ATAAA-3′) and partly on the antisense strand and highlighted in yellow (reverse complement would be 5′-GTAAA-3′). Promoter elements, -10 and -35, +1 A transcriptional start site and ribosomes binding site (RBS) were recognised as per the publication of Fang and colleagues (13).

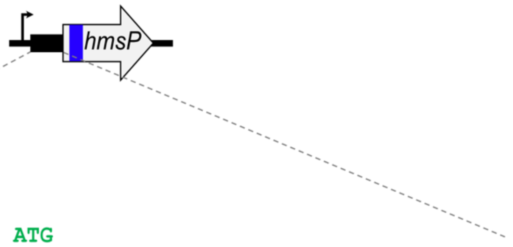


```
               +1G.........213 bp upstream to  ATG
Yptb-YPIII     TGGCACCGTCAACGTTAAGATTTCAGGGTAGGGCGGATCTGGCCTAATGATAGGGAAATG  60
               ||||||||||||||||||||||||||| ||  ||||||||||||||||||||||||||||
Ype-KIM10+     TGGCACCGTCAACGTTAAGATTTCAGGATA--GCGGATCTGGCCTAATGATAGGGAAATG  4262830

Yptb-YPIII     CGCTTAAGGGATCACAACTTACTCGATGGGGACTAAAAAGGGGCGTCTAAACACCAAGCA  120
               ||||||||||||||||||||||||||||||||||||||||||||||||||||||||||||
Ype-KIM10+     CGCTTAAGGGATCACAACTTACTCGATGGGGACTAAAAAGGGGCGTCTAAACACCAAGCA  4262890
                                                          RBS   HmsP
Yptb-YPIII     CTTGAACACCAAACGTCTGAACACCAAAAACAGTACTGCGAAATGGCGTTCCTAATAGGA  180
               ||||||||||||||||||||||||||||||||||||||||||||||||||||||||||||
Ype-KIM10+     CTTGAACACCAAACGTCTGAACACCAAAAACAGTACTGCGAAATGGCGTTCCTAATAGGA  4262950

Yptb-YPIII     AAAGACCGAAACCCACATTATTCTTGCCTTCCATTAGAGTATCGCAAGATAAAATCGGCA  240
               |||||||||||||||||||||||||||||||||||||||||||||||||||||||||| |
Ype-KIM10+     AAAGACCGAAACCCACATTATTCTTGCCTTCCATTAGAGTATCGCAAGATAAAATCGGTA  4263010

Yptb-YPIII     AGCGATCGAGTATACTCAGACAGAATCGACGAGTGGTTAGAGGATAGTAACAACCACCTT  300
               ||||||||||||||||||||||||||||||||||||||||||||||||||||||||||||
Ype-KIM10+     AGCGATCGAGTATACTCAGACAGAATCGACGAGTGGTTAGAGGATAGTAACAACCACCTT  4263070
                                                HmsP(Kegg)
Yptb-YPIII     ATTTTATGCAGTCGGAATTGGCGGAGAACGAGTTTG 333
               |||||||||||||||||||||||||||||||||
Ype-KIM10+     ATTTTATGCAGTCGGAATTGGCGGAGAACGAGT   4263103
```

Supplementary Figure 9: *Y. pseudotuberculosis hmsP* regulatory region is similar to *Y. pestis*. Shown is the genetic organisation of the *Y. pseudotuberculosis hmsP* gene. The P_{hmsP} EMSA fragment (thick horizonal black line; zoomed out DNA sequence) from the upstream promoter region of *hmsP* of *Y. pseudotuberculosis* (*Yptb*-YPIII) was aligned with *Yersinia pestis* (*Ype*-KIM10+) whole genome (sequence ID: AE009952.1) using NCBI-BLAST. P_{hmsP} EMSA fragment reveals 99% genomic identity (pair-wise alignment) between the two sequences. Non-identical nucleotides are highlighted in grey. The promoter transcribing *hmsP* is indicated by a directional arrow. ATG and TTG identify the potential start codons of *hmsP* as recognised by Fang and colleagues (13) and Kegg sequence database of *Yptb*-YPIII (https://www.genome.jp/kegg-bin/show_organism?org=ypy), respectively. A potential CpxR~P binding motif is shown in green highlight (5′-CTAAA-N_9-CTAAA-3′) and is based on similarity to the established consensus sequence of 5′-GTAAA$(N)_{4-8}$ GTAAA-3′ (11, 12). The G +1 transcriptional start site at 213 bp upstream of the ATG start codon, and the ribosome binding site (RBS) was reported by Fang and colleagues (13).

Unprocessed (raw) image of immunoblots and EMSA-gels. The area represented with a red dotted line marks the enclosed area used in the main text or supplementary figures.

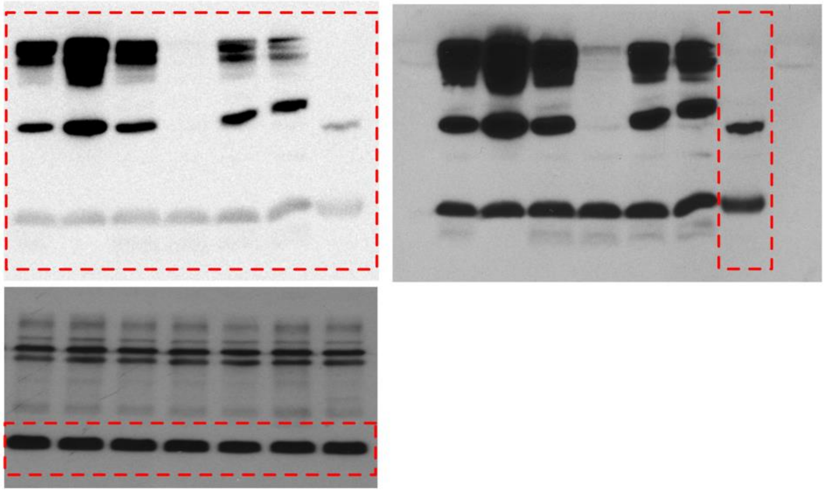

Supplementary Figure 2

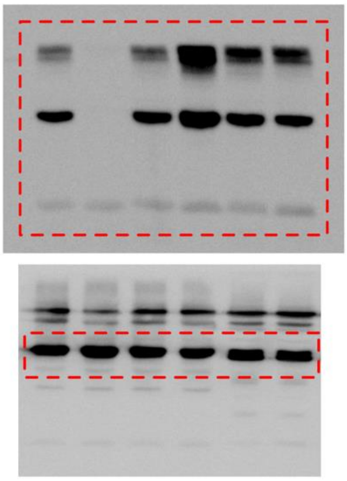

Figure 3

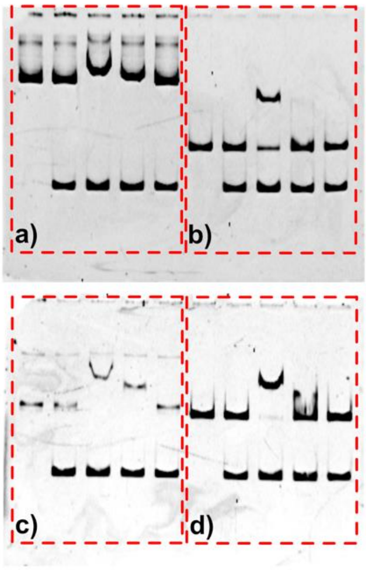

Figure 5

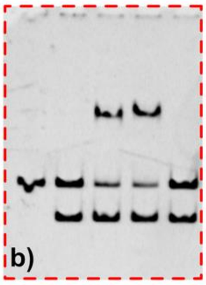

Figure 7
