# Supplementary material for: Cpx-signalling facilitates Hms-dependent biofilm formation by Yersinia pseudotuberculosis
Source: NPJ Biofilms Microbiomes. 2022 Mar 29;8:13. doi: 10.1038/s41522-022-00281-4 (PMC8964730; doi:10.1038/s41522-022-00281-4)
Supplement: Supplementary file 2 — Updated Reporting Summary. [file 41522_2022_281_MOESM2_ESM.pdf]

nature portfolio

Corresponding author(s): DBPR
Manuscript Number: NPJBIOFILMS-01478R2

Last updated by author(s): Feb 7, 2022

Reporting Summary

Nature Portfolio wishes to improve the reproducibility of the work that we publish. This form provides structure for consistency and transparency in reporting. For further information on Nature Portfolio policies, see our Editorial Policies and the Editorial Policy Checklist.

Statistics

For all statistical analyses, confirm that the following items are present in the figure legend, table legend, main text, or Methods section.

n/a	Confirmed	
☐	☒	The exact sample size (n) for each experimental group/condition, given as a discrete number and unit of measurement
☐	☒	A statement on whether measurements were taken from distinct samples or whether the same sample was measured repeatedly
☐	☒	The statistical test(s) used AND whether they are one- or two-sided<br>*Only common tests should be described solely by name; describe more complex techniques in the Methods section.*
☒	☐	A description of all covariates tested
☒	☐	A description of any assumptions or corrections, such as tests of normality and adjustment for multiple comparisons
☐	☒	A full description of the statistical parameters including central tendency (e.g. means) or other basic estimates (e.g. regression coefficient) AND variation (e.g. standard deviation) or associated estimates of uncertainty (e.g. confidence intervals)
☐	☒	For null hypothesis testing, the test statistic (e.g. F, t, r) with confidence intervals, effect sizes, degrees of freedom and P value noted<br>*Give P values as exact values whenever suitable.*
☒	☐	For Bayesian analysis, information on the choice of priors and Markov chain Monte Carlo settings
☒	☐	For hierarchical and complex designs, identification of the appropriate level for tests and full reporting of outcomes
☒	☐	Estimates of effect sizes (e.g. Cohen's d, Pearson's r), indicating how they were calculated

Our web collection on statistics for biologists contains articles on many of the points above.

Software and code

Policy information about availability of computer code

Data collection	Affinity Designer, GraphPad Prism-7, Image J, KEGG Database of Yersinia pseudotuberculosis YPIII, NCBI-BLAST, LAS4000-ImageQuant™ (GE Healthcare), Infinite-200 Tecan i-control software (version 1.12.4.0), X-Ray film, Microsoft Excel, Microsoft word and Microsoft Power-point.
Data analysis	Data analysed by above tools/softwares is properly cited and mentioned in corresponding section throughout the manuscript.

For manuscripts utilizing custom algorithms or software that are central to the research but not yet described in published literature, software must be made available to editors and reviewers. We strongly encourage code deposition in a community repository (e.g. GitHub). See the Nature Portfolio guidelines for submitting code & software for further information.

Data

Policy information about availability of data

All manuscripts must include a data availability statement. This statement should provide the following information, where applicable:

- Accession codes, unique identifiers, or web links for publicly available datasets
- A description of any restrictions on data availability
- For clinical datasets or third party data, please ensure that the statement adheres to our policy

The datasets generated and/or analysed during the current study are available from the corresponding authors on reasonable request.

Field-specific reporting

Please select the one below that is the best fit for your research. If you are not sure, read the appropriate sections before making your selection.

☒ Life sciences ☐ Behavioural & social sciences ☐ Ecological, evolutionary & environmental sciences

For a reference copy of the document with all sections, see nature.com/documents/nr-reporting-summary-flat.pdf

Life sciences study design

All studies must disclose on these points even when the disclosure is negative.

Sample size	A varied number of sample size with a minimum three biological and three technical replicates of each was used throughout the manuscript and mentioned in the respective section.
Data exclusions	A representative Image/Figure/data analysis from a minimum of three biological and three technical replicates of each was used throughout.
Replication	A minimum of three biological and three technical replicates of each were used throughout. Unprocessed (raw) blots and gels are also provided in Supplementary information.
Randomization	Not applicable
Blinding	Not applicable

Reporting for specific materials, systems and methods

We require information from authors about some types of materials, experimental systems and methods used in many studies. Here, indicate whether each material, system or method listed is relevant to your study. If you are not sure if a list item applies to your research, read the appropriate section before selecting a response.

Materials & experimental systems

n/a	Involved in the study	
☐	☒	Antibodies
☒	☐	Eukaryotic cell lines
☒	☐	Palaeontology and archaeology
☐	☒	Animals and other organisms
☒	☐	Human research participants
☒	☐	Clinical data
☒	☐	Dual use research of concern

Methods

n/a	Involved in the study	
☒	☐	ChIP-seq
☒	☐	Flow cytometry
☒	☐	MRI-based neuroimaging

Antibodies

Antibodies used	Anti-CpxR and Anti-DnaJ antibodies against Yersinia pseudotuberculosis, a mild bacterial pathogen (Agrisera AB Box 57, SE-911 21 Vännäs, SWEDEN)
Validation	Validation against total protein pool from Yersinia pseudotuberculosis and deletion mutagenesis (in case of Anti-CpxR)

Animals and other organisms

Policy information about studies involving animals; ARRIVE guidelines recommended for reporting animal research

Laboratory animals	Not applicable
Wild animals	Not applicable
Field-collected samples	Data collected/analysed from bacteria, Yersinia pseudotuberculosis (Yptb), E. coli and nematode, Caenorhabditis elegans
Ethics oversight	Not applicable

Note that full information on the approval of the study protocol must also be provided in the manuscript.
